# Metabolic and inflammatory perturbation of diabetes associated gut dysbiosis in people living with and without HIV infection

**DOI:** 10.1186/s13073-024-01336-1

**Published:** 2024-04-20

**Authors:** Kai Luo, Brandilyn A. Peters, Jee-Young Moon, Xiaonan Xue, Zheng Wang, Mykhaylo Usyk, David B. Hanna, Alan L. Landay, Michael F. Schneider, Deborah Gustafson, Kathleen M. Weber, Audrey French, Anjali Sharma, Kathryn Anastos, Tao Wang, Todd Brown, Clary B. Clish, Robert C. Kaplan, Rob Knight, Robert D. Burk, Qibin Qi

**Affiliations:** 1 Department of Epidemiology and Population Health, Albert Einstein College of Medicine, Bronx, NY USA; 2 Department of Microbiology and Immunology, Albert Einstein College of Medicine, Bronx, NY USA; 3 Department of Internal Medicine, Rush University Medical Center, Chicago, IL USA; 4 Department of Epidemiology, Johns Hopkins Bloomberg School of Public Health, Baltimore, MD USA; 5 Department of Neurology, State University of New York-Downstate Medical Center, Brooklyn, NY USA; 6 Hektoen Institute of Medicine, Chicago, IL USA; 7 Department of Medicine, Albert Einstein College of Medicine, Bronx, NY USA; 8 Department of Obstetrics and Gynecology and Women’s Health, Albert Einstein College of Medicine, Bronx, NY USA; 9 Division of Endocrinology, Diabetes, and Metabolism, Department of Medicine, Johns Hopkins University School of Medicine, Baltimore, MD USA; 10 Broad Institute of MIT and Harvard, Cambridge, MA USA; 11 Public Health Sciences Division, Fred Hutchinson Cancer Research Center, Seattle, WA USA; 12 Center for Microbiome Innovation, University of California, San Diego, La Jolla, CA USA; 13 Department of Bioengineering, University of California, San Diego, La Jolla, CA USA; 14 Department of Pediatrics, University of California, San Diego, La Jolla, CA USA; 15 Department of Computer Science and Engineering, University of California, San Diego, La Jolla, CA USA; 16 Department of Pediatrics, Albert Einstein College of Medicine, Bronx, NY USA; 17 Department of Nutrition, Harvard T.H. Chan School of Public Health, Boston, MA USA

**Keywords:** HIV infection, Diabetes, Gut dysbiosis, Gut metagenome, Inflammatory proteome, Blood metabolome, Multi-omics integration

## Abstract

**Background:**

Gut dysbiosis has been linked with both HIV infection and diabetes, but its interplay with metabolic and inflammatory responses in diabetes, particularly in the context of HIV infection, remains unclear.

**Methods:**

We first conducted a cross-sectional association analysis to characterize the gut microbial, circulating metabolite, and immune/inflammatory protein features associated with diabetes in up to 493 women (~ 146 with prevalent diabetes with 69.9% HIV +) of the Women’s Interagency HIV Study. Prospective analyses were then conducted to determine associations of identified metabolites with incident diabetes over 12 years of follow-up in 694 participants (391 women from WIHS and 303 men from the Multicenter AIDS Cohort Study; 166 incident cases were recorded) with and without HIV infection. Mediation analyses were conducted to explore whether gut bacteria–diabetes associations are explained by altered metabolites and proteins.

**Results:**

Seven gut bacterial genera were identified to be associated with diabetes (FDR-q <  0.1), with positive associations for *Shigella*, *Escherichia*, *Megasphaera*, and *Lactobacillus*, and inverse associations for *Adlercreutzia*, *Ruminococcus*, and *Intestinibacter*. Importantly, the associations of most species, especially *Adlercreutzia* and *Ruminococcus*, were largely independent of antidiabetic medications use. Meanwhile, 18 proteins and 76 metabolites, including 3 microbially derived metabolites (trimethylamine N-oxide, phenylacetylglutamine (PAGln), imidazolepropionic acid (IMP)), 50 lipids (e.g., diradylglycerols (DGs) and triradylglycerols (TGs)) and 23 non-lipid metabolites, were associated with diabetes (FDR-q <  0.1), with the majority showing positive associations and more than half of them (59/76) associated with incident diabetes. In mediation analyses, several proteins, especially interleukin-18 receptor 1 and osteoprotegerin, IMP and PAGln partially mediate the observed bacterial genera–diabetes associations, particularly for those of *Adlercreutzia* and *Escherichia*. Many diabetes-associated metabolites and proteins were altered in HIV, but no effect modification on their associations with diabetes was observed by HIV.

**Conclusion:**

Among individuals with and without HIV, multiple gut bacterial genera, blood metabolites, and proinflammatory proteins were associated with diabetes. The observed mediated effects by metabolites and proteins in genera–diabetes associations highlighted the potential involvement of inflammatory and metabolic perturbations in the link between gut dysbiosis and diabetes in the context of HIV infection.

**Supplementary Information:**

The online version contains supplementary material available at 10.1186/s13073-024-01336-1.

## Background

Diabetes, mainly type 2 diabetes, is a prevailing metabolic disorder, posing a significant public health challenge worldwide. People living with human immunodeficiency virus (HIV) infection (PLWH) are at high risk of metabolic diseases, including diabetes, possibly due to the chronic inflammation and persistent immune activation induced by HIV infection and the long-term antiretroviral therapy (ART) [1]. Characterizing modifiable risk factors of diabetes beyond traditional risk factors is thus essential to the diabetes prevention and management in this susceptible population.

Emerging evidence suggests that gut dysbiosis may play a critical role in the development of diabetes [2–4]. Both our pilot study and other prior work have reported altered microbiota composition associated with some metabolic disorders, such as metabolic syndrome [5], prediabetes [6], and diabetes [7] among PLWH. However, these previous studies were limited by relatively small sample sizes, and characteristics of gut dysbiosis associated with diabetes in the context of HIV infection are not fully understood. How and through which molecular mechanisms gut dysbiosis may contribute to diabetes in HIV infection remains largely unclear. Evidence from experimental settings has suggested that gut microbiota may affect the host metabolic health through direct modulation of host immunity/inflammation responses [2, 4, 8], as well as by producing microbial-derived biomolecules especially those play a role in modulation of host metabolism, inflammation, and immune activation (e.g., indoles, short-chain fatty acids [SCFAs], secondary bile acids) [9, 10]. Given that alterations in gut microbial taxonomic composition (e.g., the loss of beneficial microbes and enriched pathobionts) and related microbial functionalities, as well as disrupted inflammation and immune activation homeostasis, have been noted in HIV infection [11–14], we thus hypothesized that among PLWH or population at high risk for HIV infection, gut dysbiosis is associated with diabetes, which is partially explained by metabolic-inflammatory perturbations.

To test this hypothesis, we leveraged multi-omics data from the Women’s Interagency HIV Study (WIHS) and the Multicenter AIDS Cohort Study (MACS), two well-characterized cohorts of both PLWH and demographically and socioeconomically similar people without HIV infection in US, to first identify gut microbial taxonomic features, circulating metabolites, immune and inflammatory proteins associated with diabetes, and then to explore the potential mediating roles of metabolites and inflammatory proteins in the observed gut microbiota–diabetes relationship.

## Methods

### Study design and population

The WIHS was a multicenter longitudinal study started in 1993 and designed to investigate the long-term, natural, treated history, and progression of HIV infection in women [15]. The MACS was a prospective study originally founded in 1984 and focused on the natural and treated histories of HIV infection in men [16]. The WIHS and MACS used similar study designs and methods, recruited both PLWH and HIV-negative people with similar demographical and socioeconomical status and high-risk behaviors for HIV infection, and were merged to form the MACS-WIHS Combined Cohort Study in the beginning of 2019 [17]. In the present investigation, we included 563 women who provided stool samples from three sites (Bronx, Brooklyn, and Chicago) during core visits at 6-month intervals from 2017 to 2019 in WIHS [18, 19]. Among them, 493 women had shotgun metagenomics profiled, 434 had plasma metabolomics profiled, and 428 had serum immune/inflammatory proteins. Among 426 participants with all these three omics data, 396 (~ 93%) had the omics data measured on biospecimens collected at the same visits and 12 had data matched within 1 year (i.e., ≤ 2 visits). To examine the prospective relationship between plasma metabolites and incident diabetes, we included 694 participants (391 women from WIHS and 303 men from MACS; 166 incident cases were recorded over a median follow-up of 12.6 years) who were free of diabetes and with metabolomic and lipidomic data at a baseline visit (2004–2006) [20, 21]. An overview of study design and sample selection of the present study is shown in Fig S1.

### Gut* metagenomics profiling*

In this study, only participants from WIHS provided fecal samples for gut metagenomics profiling. Fecal samples were collected using a home-based self-collection kit that was distributed to each participant at a core WIHS visits as previously described [18, 19]. Metagenomic sequencing was performed on DNA extracted from fecal samples using a shallow-coverage method of shotgun sequencing-based Illumina NovaSeq platform [19]. De-multiplexing was applied to generate per-sample FASTQ data, which was further trimmed to remove low-quality bases with a 25 or less PHRED quality score using prinseq-lite 0.20.4 (https://edwards.sdsu.edu/cgibin/prinseq/prinseq.cgi). After quality control, FASTQ data were then concatenated and aligned to the NCBI RefSeq representative prokaryotic genome collection using the SHOGUN pipeline (https://github.com/knights-lab/SHOGUN), through which reads were labeled with NCBI taxonomic annotation at the species level.

### Plasma metabolomics profiling

Metabolomics profiling was performed on plasma samples using hydrophilic interaction liquid chromatography/positive ion mode (HILIC-pos for water soluble metabolites) and reversed-phase C8 chromatography/positive ion mode mass spectrometry (C8-pos for liposoluble metabolites) at the Broad Institute Metabolomics Platform (Cambridge, Massachusetts) as previously described [21]. In this study, we included 378 metabolites (211 lipids and 167 nonlipids) with detection rate > 75% in cross-sectional analyses of prevalent diabetes involving women from WIHS and 325 metabolites (211 lipids and 114 nonlipids) in the prospective analyses of incident diabetes involving both women from WIHS and men from MACS. Levels of metabolites below detection were replaced by one half of the minimum detected values.

### Assessment of inflammatory proteins

A set of inflammation-related proteins were quantified in serum among 428 women from WIHS using the Olink Inflammation panel (92 proteins, Olink Bioscience, Uppsala, Sweden). Data of targeted proteins is presented as normalized protein expression (NPX) values with the unit at log2 scale. We included 74 proteins detected in > 75% of samples and with average coefficient of variance (CV, %) < 10% (intra-assay CV = 4%, inter-assay CV = 9%). Levels of proteins below detection were replaced by one half of the square root of the limit of detection.

### Ascertainment of HIV infection, diabetes, and other covariates

Data on sociodemographic, behavioral, and clinical characteristics, including laboratory testing, were collected following standardized protocols [15, 16, 21]. Medication histories including use of antihypertensive, lipid-lowering, and antidiabetic medications were collected with standardized interviewer-administered questionnaires. HIV serostatus was ascertained by enzyme-linked immunosorbent assay and confirmed by Western blot. Other HIV-related parameters included serum CD4 + cell counts, HIV RNA viral load, and current use of ART drugs. Diabetes was defined as fasting plasma glucose ≥ 126 mg/dL, random plasma glucose ≥ 200 mg/dL, HbA1c ≥ 6.5%, or self-report use of antidiabetic medications in line with our previous work [21]. In the prospective analysis, participants who were free of diabetes at baseline but developed diabetes during follow-up were defined as incident diabetes cases.

### Statisticalanalysis

The Analysis of Composition of Microbiome-II (ANCOM-II) [22] method that accounts for structural zeros was used to identify gut bacteria associated with prevalent diabetes among 493 women in WIHS. ANCOM-II was conducted at both the genus (*n* =  97) and species (*n* =  203) levels with predominant bacterial taxa (relative abundance > 0.01% and prevalence > 10%) included, while adjusting for age, study site, race/ethnicity, household annual income, education, smoking, alcohol consumption, HIV serostatus, and antibiotic use within 4 weeks of stool sample collection. The false discovery rate (FDR) using the Benjamini–Hochberg method was controlled at 0.1, and the threshold of identifying significant microbiota taxa in ANCOM-II was set at 0.6, which refers to ratio of the tested taxon to at least 60% of the other taxa detected to be significantly associated with diabetes. The associations of identified gut bacterial taxa (centered log-ratio transformed, CLR) with prevalent diabetes were further examined using logistic regression with the adjustment of the same set of covariates. Additionally, differences in bacterial α-diversity at the species level (observed, Chao1, Shannon, and Simpson diversity indices) between women with and without diabetes were tested by the Kruskal–Wallis test. Association between β diversity (Bray–Curtis dissimilarity) and diabetes was tested using the permutational multivariate analysis of variance (PERMANOVA, *n* =  9999 permutations) [23].

The cross-sectional associations of serum proteins (*N* =  428, inverse normal transformed (INT)) and metabolites (*N* =  434, INT) with prevalent diabetes in WIHS women were assessed using logistic regression models adjusted for age, study site, race/ethnicity, household annual income, education, smoking, HIV serostatus, and fasting status. The false discovery rate was controlled at 0.1. For the identified proteins, we conducted sparse partial least square discriminant analysis (sPLS-DA) [24] to examine their performance in classifying participants with and without diabetes. In addition, we used Cox proportional hazards models to examine prospective associations of identified metabolites with incident diabetes among 694 women and men who were free of diabetes at baseline, adjusting for sex and the same set of covariates in the aforementioned cross-sectional analysis. Analyses were also conducted in women and men separately and results were then combined by meta-analysis to test potential sex differences in metabolite–incident diabetes associations. To reduce the dimension of the diabetes-associated metabolites, the weighted correlation network analysis (WGCNA) [25] was applied to identify metabolite modules for downstream analysis.

After omics-wide association analyses, we conducted partial correlation analyses, adjusting for the covariates listed above, to assess the interrelationship among identified diabetes-associated gut bacteria, proteins, and metabolites. By using the “cmest” function from the R CMAverse package [26], mediation analyses were conducted to investigate whether associations between gut bacteria and diabetes were partially mediated by alterations in circulating metabolites and proteins. Details about this mediation analysis were provided in the Supplementary Methods.

To test the robustness of our results for omics–diabetes associations, we further adjusted for a set of diabetes-related factors, including body mass index (BMI), triglycerides, systolic blood pressure (SBP), high-density lipoprotein cholesterol (HDL-C), and use of lipid lowering and antihypertensive medications. Given that diabetes treatments (e.g., metformin) have been linked with gut microbiota composition [27–29], we further conducted a “drug deconfounding” analysis [28, 29] using a recently introduced two-step pipeline to examine whether the identified gut microbiota–diabetes associations were confounded by antidiabetic medications. A brief introduction to this “drug deconfounding” analysis is provided in the Supplementary Methods. Stratified analyses on the associations between the identified omics features and diabetes were also conducted by HIV serostatus. To test potential effect modification by HIV serostatus, we included a product term with omics features in the regression models. Differences in the levels of identified diabetes-associated omics features between women with and without HIV infection were also compared using analysis of covariance (ANCOVA). To further integrate HIV infection, gut dysbiosis, and metabolic and inflammatory perturbation with diabetes, we conducted in parallel omics wide association analyses to characterize omics features associated with HIV infection and evaluate their associations with diabetes.

In the above association analyses, all omics features were standardized into Z-scores after the original transformation (CLR for microbial features and INT for metabolites/proteins) to improve the comparability of each feature in association with diabetes. All analyses were conducted using R 3.4. Statistical significance was set at 0.05 (two-tailed) unless otherwise stated.

## Results

### Population characteristics

As shown in Table S1, up to 493 women, including 336 living with HIV, with a mean age of 53.2 years, were included in the cross-sectional, omics-wide association analysis of prevalent diabetes. Approximately 91% of women living with HIV reported receiving ART drugs within 6 months at the time of stool samples collection and nearly 73.5% had undetectable HIV viral load. Compared to women without diabetes, women with diabetes tended to be older and had an unfavorable profile of cardiometabolic traits and higher proportion of antihypertensive and lipid lowering medication use.

### Gut* microbiota and diabetes*

**Fig. 1:**
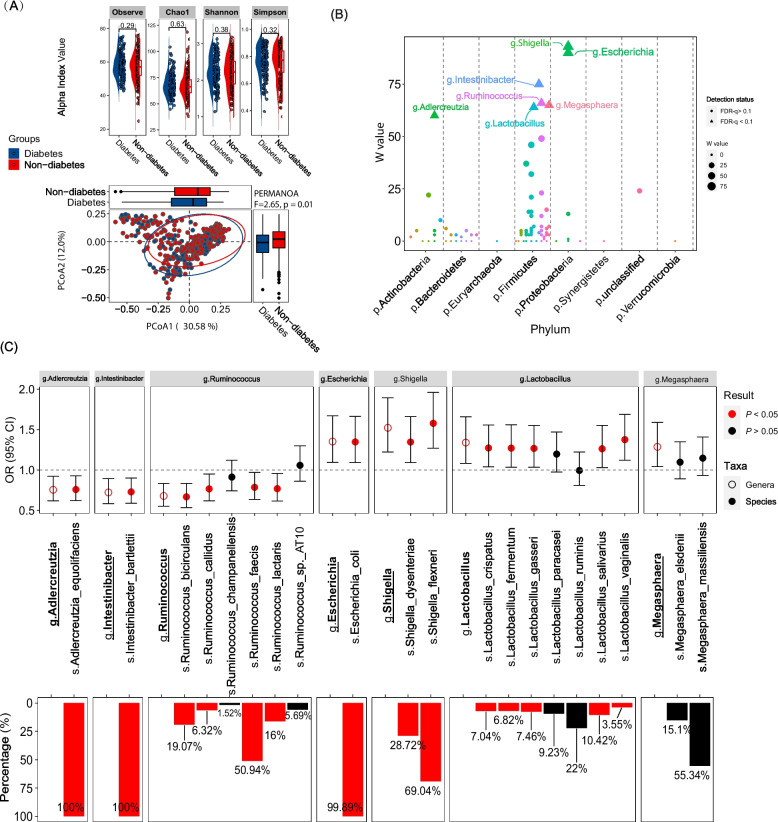
Gut microbiota composition and prevalent diabetes (*N* = 493). A Differences in alpha and beta diversities of gut microbiota at the species level between women with and without diabetes. The Wilcoxon rank test was used for the comparison of alpha diversity (observe, Chao1, Shannon, and Simpson indices). Bray–Curtis dissimilarity was used to calculate beta diversity, which was represented by the first two components of principal coordinates analysis (PCoA). Difference in beta diversity across diabetes status was tested using permutational multivariate analysis of variance (PERMANOVA with 9999 permutations). B ANCOM-II results of the 97 predominant gut bacteria genera. Genera marked as triangles were those associated with diabetes with FDR-q < 0.1 at threshold of 0.60 (i.e., the ratio of genera to at least 60% of the other taxa is detected to be significantly associated with diabetes). Color refers to phyla and sizes refer to W values in ANCOM-II. The prefixes of “g.” and “s.” refer to the taxa at genus and species levels, respectively. C Associations (odds ratio (ORs) and 95% confidential intervals (CIs)) of identified gut bacteria genera and affiliated species with diabetes (top panel) and the percentages of species within selected genera (bottom panel). Species presented in ≥5% of samples with a relative abundance ≥0.01% were included. Estimates in B and C were adjusted for age at visit, study site, race/ethnicity, household annual income, education, smoking, alcohol consumption, HIV serostatus, and antibiotics use within the 4 weeks of stool sample collections

We found a significant difference in β-diversity (PMERANOVA, *P* = 0.01) but not in α-diversity between women with and without diabetes (Fig. 1A). ANCOM-II at the genus level identified 7 out of 97 predominant bacteria genera associated with diabetes (FDR-*q* < 0.1), including 4 *Firmicutes* genera (i.e., *Intestinibacter*, *Ruminococcus*, *Megasphaera*, and *Lactobacillus*), 2 *Proteobacteria* genera (i.e., *Shigella* and *Escherichia*), and 1 *Actinobacteria* genus (*Adlercreutzia*) (Fig. 1B and Table S2). Specifically, *Adlercreutzia*, *Intestinibacter*, and *Ruminococcus* were inversely associated with diabetes, whereas *Shigella, Escherichia, Megasphaera*, and *Lactobacillus* exhibited positive associations with diabetes (Fig. 1C). We identified 20 common species (relative abundance > 0.01% and prevalence > 5%) within these 7 genera and found that most species within the same genus showed generally consistent associations with diabetes (Fig. 1C). Consistently, species within the same genus were positively correlated with each other (Fig S2). We also found strong positive correlations between members of *Shigella* and *Escherichia*, the two bacteria in the *Enterobacteriaceae* family, at both the genus and species levels. ANCOM-II at the species levels only identified one additional species that was associated with diabetes, *Eubacterium_eligens*, which does not belong to any of these 7 genera (Fig S3). Given these observations, we thus focused on the identified diabetes-associated genera in the following analyses.

Further adjustment of major diabetes-related metabolic traits (BMI, triglycerides, HDL, and SBP) and use of antihypertensive and lipid-lowering medications did not substantially change the associations of these bacterial genera with diabetes (Table S3). The “drug deconfounding” analysis suggested that associations of *Intestinibacter* and *Escherichia* with diabetes might be confounded by antidiabetic medication use (Fig S4A). For example, women with treated diabetes had significantly higher abundance of *Escherichia* compared to women without diabetes and those with untreated diabetes, whereas abundance of this genus was similar between women without diabetes and those with untreated diabetes (Fig S4B). A similar pattern was found for *Shigella* (Fig S4B).

### Serum inflammatory proteins and diabetes

**Fig. 2:**
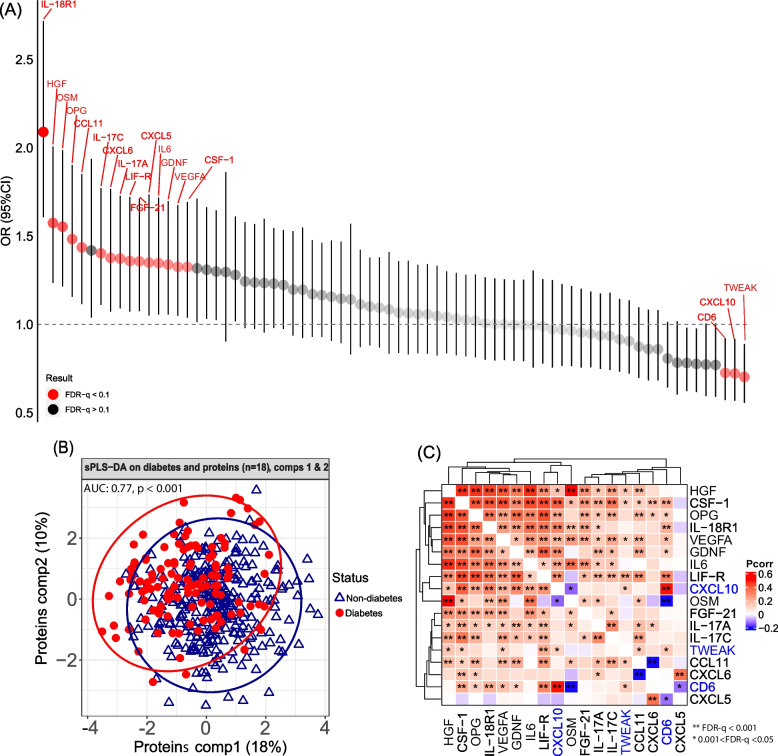
Inflammatory proteomics and prevalent diabetes (*N* = 428). **A** Associations (odds ratio (OR) and 95% confidence interval (CI)) between inflammatory proteins (*n* = 74) and prevalent diabetes in binary logistic regression. Significant proteins (*n* = 18, FDR-*q* < 0.1) were labeled in red. **B** Representations of diabetes status by the first two components of diabetes-associated proteins (*n* = 18) in sparse partial least squares regression for discrimination analysis (sPLS-DA). **C** Partial spearman correlation (Pcorr) among the 18 diabetes-associated proteins. Proteins inversely associated with diabetes were labeled in blue. Analyses of logistic regression, partial correlation, and sPLS-DA were adjusted for age at visit, study site, race/ethnicity, household annual income, education, smoking, alcohol consumption, HIV serostatus, and fasting status. CXCL11: C-X-C motif chemokine 11, CD6: T cell surface glycoprotein CD6 isoform, CSF-1: Macrophage colony-stimulating factor 1, CXCL10: C-X-C motif chemokine 10, CXCL5: C-X-C motif chemokine 5, CXCL6: C-X-C motif chemokine 6, FGF-21: fibroblast growth factor 21, GDNF: glial cell line-derived neurotrophic factor, HGF: hepatocyte growth factor, IL-17A: interleukin-17A, IL-17C: interleukin-17C, IL-18R1: interleukin-18 receptor 1, IL6: interleukin-6, LIF-R: leukemia inhibitory factor receptor, OPG: osteoprotegerin, OSM: oncostatin-M, TWEAK: tumor necrosis factor (ligand) superfamily, member 12, VEGFA: vascular endothelial growth factor A

After multivariable adjustment (see “ Methods”), we found 18 out of 74 proteins associated with prevalent diabetes (FDR-q < 0.1), with inverse associations for T cell surface glycoprotein CD6 isoform (CD6), C-X-C motif chemokine 10 (CXCL10), and tumor necrosis factor (ligand) superfamily, member 12 (TWEAK), and positive associations for the remaining 15 proteins (Fig.2A). Interleukin-18 receptor 1 (IL-18R1) showed the strongest positive association with diabetes (OR =  2.09 [95%CI: 1.61, 2.73] per SD increase), while TWEAK showed the strongest inverse association with diabetes (OR =  0.70 [95%CI: 0.56, 0.89]). Associations of these 18 proteins with diabetes did not change appreciably when we adjusted for diabetes-related metabolic traits and use of anti-hypertensive and lipid-lowering medications (Table S4). Using sPLS-DA, we found that the first two components of these identified 18 proteins generally had a good performance in diabetes classification (AUC = 0.77, *P* < 0.001).

Partial correlation analysis revealed a cluster of seven proteins (hepatocyte growth factor (HGF), macrophage colony-stimulating factor 1 (CSF-1), osteoprotegerin (OPG), IL-18R1, vascular endothelial growth factor A (VEGFA), glial cell line-derived neurotrophic factor (GDNF), and IL6) that were highly intercorrelated (Fig. 2C) and all positively associated with diabetes (Fig. 2A). These clustered 7 proteins also showed some weak-to-moderate positive correlations with the remaining 11 proteins, including 3 proteins that were inversely associated with diabetes (CD6, CXCL10, and TWEAK). However, correlation among these 11 remaining proteins were comparatively weak.

### Plasma metabolites and diabetes

**Fig. 3:**
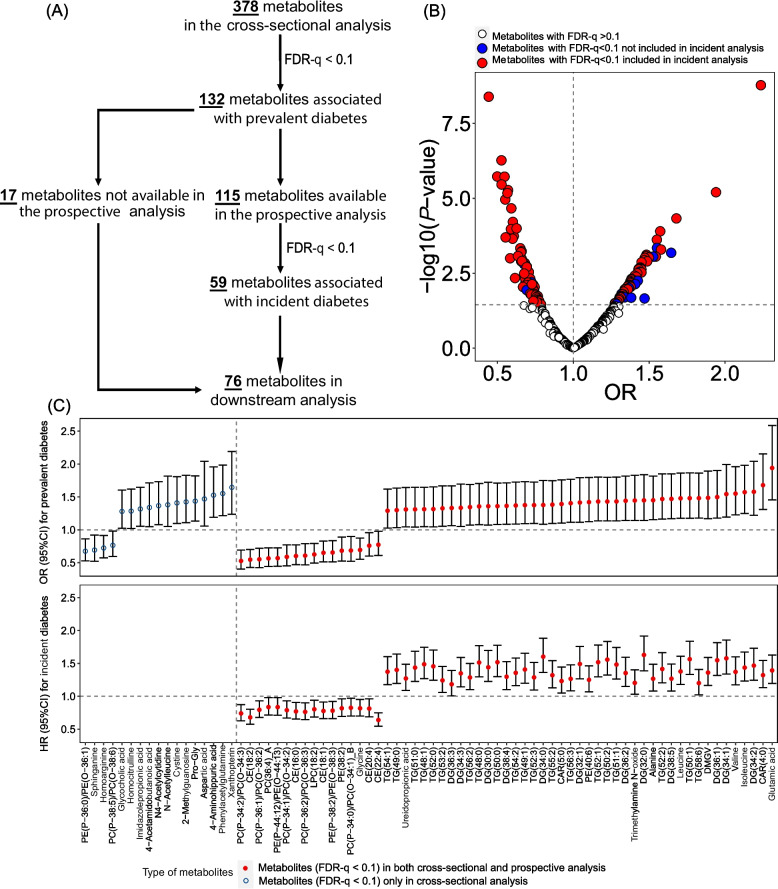
Associations between plasma metabolites and prevalent diabetes (*N* = 434). **A** An overview of analysis pipeline and study samples. **B** The distribution of cross-sectional associations between metabolites and diabetes in logistic regression. Metabolites in red and blue refer to those exhibiting positive and negative associations (FDR-q <0.1) with prevalent diabetes, respectively; among them, those colored in red were available in the prospective analysis of incident diabetes (*N* = 694, including 391 women from the Women’s Interagency HIV Study and 303 men from the Multicenter AIDS Cohort Study). **C** Significant associations with diabetes for metabolites selected in the cross-sectional analyses and those validated in the incident diabetes analysis. Estimates (odds ratios (ORs)/hazard ratios (HR)) were adjusted for age at visit, study site, race/ethnicity, household annual income, education, smoking, alcohol consumption status, HIV serostatus, and fasting status. Pro-Gly: dipeptide prolyl-glycine, DMGV: dimethylguanidino valerate

We first conducted a cross-sectional metabolome wide association analysis in 434 WIHS women and identified 132 (FDR-*q* < 0.1) out of 378 metabolites associated with diabetes (Fig. 3A). Among these 132 metabolites, 115 were available in a prospective dataset of women and men in the WIHS and MACS (*N* =  694, including 166 incident diabetes cases over a median follow-up of 12.6 years). We then conducted a prospective analysis and found that 59 (50 lipid species and 9 nonlipid metabolites) out of 115 metabolites were associated with incident diabetes (FDR-*q* < 0.10) (Fig. 3A and B). Separate analyses in women (WIHS) and men (MACS) revealed that the associations of the majority of these 59 metabolites with incident diabetes were comparable between sexes (cohorts) (Fig S5), and combined results from women and men by meta-analysis were very similar to those from the analyses in the overall sample (Table S5). Together with the 17 metabolites that were not available in the prospective dataset, a total of 76 metabolites, including 50 lipids and 26 nonlipid metabolites, were considered as diabetes-associated metabolites in the present study (Fig. 3A).

**Fig. 4:**
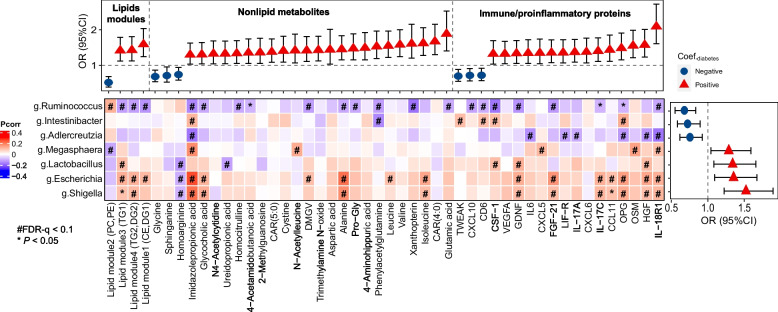
The relationship between diabetes-associated metabolites and immune/proinflammatory proteins and the identified gut bacteria associated with diabetes. Heatmap presents the partial correlation coefficients (PCorr) of gut bacteria genera with selected metabolites and proteins while adjusting for age at visit, study sites, race/ethnicity, household annual income, education, smoking, alcohol consumption, HIV serostatus, fasting status, and antibiotics use. The top forest plot displays cross-sectional associations between identified metabolites/lipids modules (FDR-q < 0.1) and diabetes; the right forest plot displays cross-sectional associations of identified bacterial genera with prevalent diabetes. In these two panels, the color refers to the direction of association. CXCL11: C-X-C motif chemokine 11, CD6: T cell surface glycoprotein CD6 isoform, CSF-1: macrophage colony-stimulating factor 1, CXCL10: C-X-C motif chemokine 10, CXCL5: C-X-C motif chemokine 5, CXCL6: C-X-C motif chemokine 6, FGF-21: fibroblast growth factor 21, GDNF: glial cell line-derived neurotrophic factor, HGF: hepatocyte growth factor, IL-17A: interleukin-17A, IL-17C: interleukin-17C, IL-18R1: interleukin-18 receptor 1, IL6: interleukin-6, LIF-R: leukemia inhibitory factor receptor, OPG: osteoprotegerin, OSM: oncostatin-M, TWEAK: tumor necrosis factor (ligand) superfamily, member 12, VEGFA: vascular endothelial growth factor A

In line with our previous findings [21], we found that lipid species belonging to diradylglycerols (DGs, *n* =  12) and triradylglycerols (TGs, *n* =  20), together with two carnitines (CAR 5:0 and CAR 4:0), were positively associated with prevalent diabetes, whereas cholesterol esters (CEs, *n* =  5) and phospholipids belonging to phosphatidylethanolamines (PEs, *n* =  5), phosphatidylcholines (PCs, *n* =  7), and one lyso-phosphatidylcholines (LPC 18:2) exhibited inverse associations with diabetes (Fig. 3C and Table S4). Correlation analysis revealed that these 50 lipids were highly intercorrelated (Fig S6) and clustered into 4 modules in WGCNA models (Fig S7), which were then included in the following analyses to reduce the dimensionality of diabetes-associated lipids. As expected, lipid modules characterized by high levels of DGs and/or TGs (i.e., lipid modules 1, 3, and 4) were positively associated with diabetes, whereas a lipid module characterized by high levels of PCs and PEs (lipid module 2) was inversely associated with diabetes (Fig. 4 and Table S4). Regarding the 26 nonlipid metabolites, 3 metabolites (glycine, homoarginine, and sphinganine) were inversely associated with diabetes, whereas positive associations with diabetes were found for the remaining 18 metabolites, including 3 microbial metabolites (trimethylamine N-oxide (TMAO) [30], phenylacetylglutamine (PAGln) [31], and imidazolepropionic acid (IMP) [32]), 3 branched-chain amino acids (BCAAs: valine, leucine, and isoleucine) and 1 related metabolite (N-Acetylleucine), 8 non-essential amino acids and related metabolites (e.g., glutamic acid and alanine), and 6 xenobiotics (e.g., xanthopterin). After further adjusting for diabetes-related metabolic traits, the associations of these metabolites/lipid modules with prevalent diabetes were largely attenuated, especially for lipids and lipid modules, though most of the associations remained statistically significant (Table S4).

### Gut microbiota, circulating metabolites, proteins, and diabetes

Partial correlation analysis revealed that the identified diabetes-associated bacteria genera were correlated with multiple diabetes-associated metabolites, lipid modules and proteins (FDR-*q* < 0.1) (Fig. 4 and Fig S8). Overall, *Ruminococcus* and *Adlercreutzia*, two genera inversely associated with diabetes, tended to be inversely correlated with metabolites and lipids modules and proteins that were positively associated with diabetes. By contrast, an overall opposite pattern with respect to the correlations with metabolites, lipid modules and proteins were found for the 4 genera (*Megasphaera*, *Lactobacillus,* *Shigella*, and *Escherichia*) that were positively associated with diabetes (Fig. 4). In particular, 6 out of 7 diabetes-associated genera (*Intestinibacter, Megasphaera, Shigella, Escherichia, Ruminococcus*, and *Adlercreutzia*) were correlated with IMP (FDR-*q* < 0.1), with inverse associations for *Ruminococcus* and *Adlercreutzia* and positive association for other 4 bacterial genera. In addition, *Ruminococcus* and *Intestinibacter* were inversely correlated with PAGln (FDR-*q* <  0.1).

**Fig. 5:**
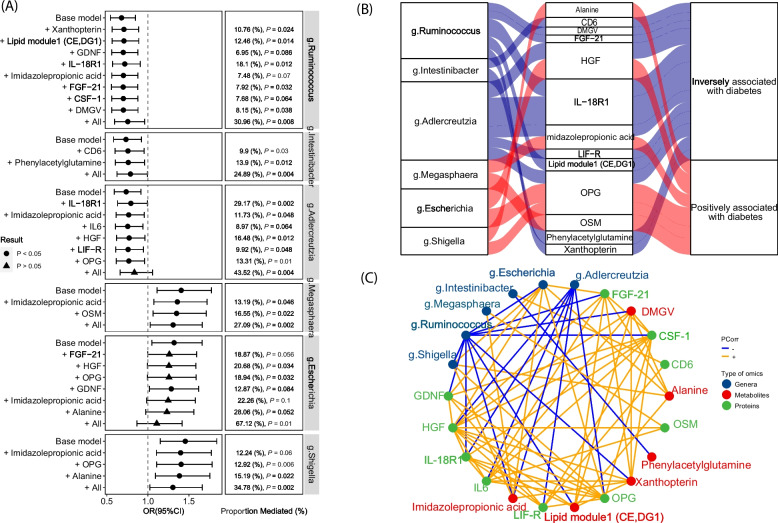
Triangular relationship among diabetes-associated microbial genera, metabolites, proteins, and prevalent diabetes (*N* = 426). **A** Mediated association of gut bacterial genera with prevalent diabetes by selected metabolites (or lipid modules) and proteins. The left forest plot shows associations of selected genera with diabetes following consecutive adjustment for selected metabolites/proteins in logistic regression. The “base model” refers to the model adjusted for age at visit, study site, race/ethnicity, household annual income, education, smoking, alcohol consumption, HIV serostatus, fasting status, and antibiotics use. “+ metabolites/proteins” refer to the models further adjusted for specific metabolites/proteins in addition to variables in the base model, while “+ All” refer to the model further simultaneously adjusted for all selected metabolites/proteins. The right panel shows proportions mediated (calculated as the ratio of indirect effects to total effects) of examined metabolites (or lipid modules) and proteins in regression-based mediation analysis (see “ Methods”). **B** An alluvium plot summarizing significant mediation effects of individual proteins and metabolites in the association between bacteria and diabetes (*P* < 0.05). “Blue” alluvia belts refer to the potential pathway indicating the negative associations of bacteria with diabetes, while “red” ones refer to pathways exhibiting positive associations of bacteria with diabetes. Size of alluvium flow refers to the relative magnitude of mediated proportions. **C** A network showing the interrelationship (presented as partial correlation coefficients) among omics signatures (genera: marked as blue; metabolites: red; proteins: green) in associations with prevalent diabetes. The colors of lines refer to the direction of correlation (positive: orange; negative: blue). For interrelationship among signatures within each omics class, only |partial coefficient|> >0.25 with FDR-*q* < 0.1 were presented. GDNF: glial cell line-derived neurotrophic factor, HGF: hepatocyte growth factor, IL-18R1: interleukin-18 receptor 1, IL6: interleukin-6, LIF-R: leukemia inhibitory factor receptor, OPG: osteoprotegerin, OSM: oncostatin-M, CD6: T cell surface glycoprotein CD6 isoform, CSF-1: macrophage colony-stimulating factor 1, FGF-21: fibroblast growth factor 21, DMGV: dimethylguanidino valerate

To test whether the associations between identified bacterial genera and diabetes can be partially explained by alterations in the identified diabetes-associated metabolites and proteins, we conducted conditional analysis and mediation analysis. Herein, we focused on metabolites, lipid modules, and proteins that have concordant associations with both diabetes and bacteria genera (FDR-*q* <  0.1, Table S 6). In conditional analyses, associations between bacteria genera and diabetes were generally attenuated when selected metabolites and proteins were included in the models (Fig. 5A). Mediation analysis revealed that the selected multiple nonlipid metabolites, lipid modules, and proteins collectively mediated a considerable proportion of the associations of 6 genera (*Intestinibacter* (proportion of mediated effect for multiple mediators =  24.89%), *Megasphaera* (27.09%), *Ruminococcus* (30.96%), *Shigella* (34.78%), *Adlercreutzia* (43.52%), and *Escherichia* (67.12%), all *P* < 0.01) with diabetes. Notably, associations of *Ruminococcus*, *Adlercreutzia*, and *Escherichia* with diabetes were mediated considerably by multiple inflammatory proteins led by OPG, IL-18R1, and HGF (Fig. 5A and B). IMP appeared to partially mediate the associations of the most identified genera with diabetes (mediated proportion ranges from 7.5 to 22.3%), though some of mediated effects were nominally significant (Fig. 5A). In addition, PAGln significantly mediated 13.9% of the inverse association between *Intestinibacter* and diabetes (Fig. 5A). The interrelationship among identified diabetes-associated bacteria, proteins, and metabolites were shown in Fig. 5C.

In addition, we also examined correlations of these omics features with glycemic traits and found that metabolites (e.g., alanine and DMGV) or proteins (e.g., OSM, IL-18R1, and HGF) significantly mediated the observed gut bacteria and diabetes associations were generally associated with examined glycemic traits in the same direction of their association with diabetes (Fig S9).

### HIV infection and diabetes-associated omics features

Stratified analyses by HIV serostatus showed generally similar associations between identified omics features and diabetes among women with and without HIV (Table S7). We then compared levels of the diabetes-associated omics features between women with and without HIV and found that multiple lipids belonging to DGs/TGs, nonlipids metabolites (e.g., IMP, PAGln, ureidopropionic acid, xanthopterin, 4-aminohippuric acid, N4-acetylcytidine, 4-acetamidobutanoic acid, glycocholic acid, and homocitrulline), and proteins (GDNF and leukemia inhibitory factor receptor (LIF-R)) that were positively associated with diabetes were significantly higher among women with HIV, especially those with detectable HIV RNA viral load (> 20 copies/mL) as compared with women without HIV (Table S8). In addition, several metabolites that were inversely associated with diabetes (e.g., homoarginine and lipids belonging to PCs/PEs/CEs) were lower in women with HIV compared with those without HIV (Table S8). However, abundances of identified diabetes-associated genera were similar among women with and without HIV (Table S8).

**Fig. 6:**
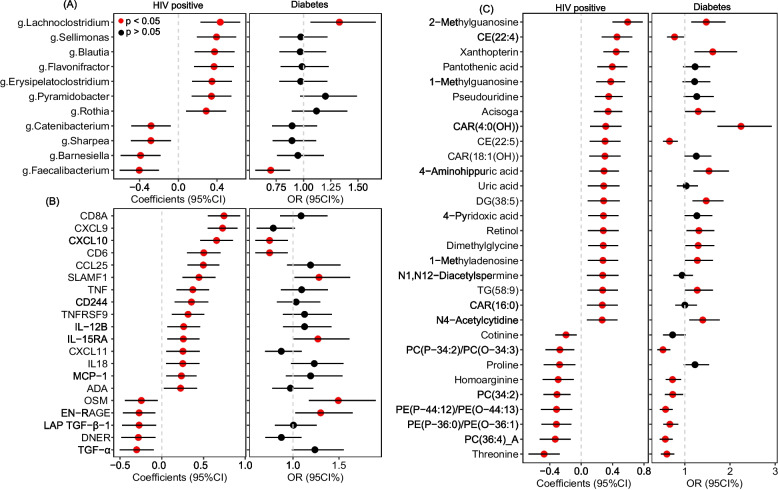
HIV infection-associated gut bacteria, proteins and metabolites and prevalent diabetes in WIHS women (*N* = 426). Data were associations and 95% confidence intervals of selected gut bacteria genera (**A**), proteins (**B**), and metabolites (**C**) with HIV infection (i.e., positive serostatus) and prevalent diabetes. Associations were derived via multivariable logistic regression models adjusting for age at visit, study site, race/ethnicity, annual household income, education, smoking, alcohol consumption, fasting status, antibiotics use (for gut bacterial features), and HIV serostatus (for association with prevalent diabetes). Only omics features significantly associated HIV infection at FDR-*q* < 0.1 were included here. Detailed numeric results were shown in Table S9. Detailed numeric results and feature annotations were shown in Table S9. CD8A: T-cell surface glycoprotein CD8 alpha chain, CXCL9: C-X-C motif chemokine 9, CXCL10: C-X-C motif chemokine 10, CCL25: C–C motif chemokine 25, SLAMF1: signaling lymphocytic activation molecule, TNF: tumor necrosis factor, CD244: natural killer cell receptor 2B4, TNFRSF9: tumor necrosis factor receptor superfamily member 9, IL-12B: interleukin-12 subunit beta, IL-15RA: interleukin-15 receptor subunit alpha, CXCL11: C-X-C motif chemokine 11, IL18: interleukin-18 (IL-18), MCP-1: monocyte chemotactic protein 1, ADA: adenosine deaminase, EN-RAGE: protein S100-A12, LAP TGF-β-1: latency-associated peptide transforming growth factor beta-1, DNER: delta and Notch-like epidermal growth factor-related receptor, TGF-α : transforming growth factor alpha

Omics-wide associations identified 11 bacterial genera (Fig. 6A), 20 proteins (Fig. 6B), and 30 metabolites (Fig. 6C) significantly associated with HIV infection (Fig. 6 and Table S9, FDR-*q* <  0.1). Of note, the majority of identified metabolites and bacteria genera had concordant associations with HIV infection and prevalent diabetes (i.e., metabolites positively/inversely associated with HIV infection were also positively/inversely associated with diabetes (Fig S10), including two bacteria (e.g., *Lanchoclostridum* and *Faecalibacterium*) and 11 nonlipids metabolites exhibiting significant associations with diabetes (Fig. 6A and C). In addition, we found two proteins significantly associated with both HIV and diabetes in the same direction (Fig. 6B), but many of these identified HIV-associated proteins were not associated with diabetes.

## Discussion

In the present study, among PLWH and demographically and socioeconomically similar individuals without HIV infection, we characterized multi-omics features associated with prevalent diabetes, including 7 gut bacterial genera, 18 immune and pro-inflammatory proteins, and 76 metabolites. More than half of metabolites associated with prevalent diabetes were confirmed to be associated with incident diabetes over a median follow-up of 12 years in a prospective cohort. Moreover, several proteins, especially IL-18R1 and OPG, and microbial derived metabolites, including IMP and PAGln, significantly mediated the associations between identified gut bacteria and diabetes, both individually and in combination.

Our findings on the observed altered gut microbiota composition associated with diabetes are generally consistent with findings among general (i.e., non-HIV) populations, including the positive associations of *Shigella* [2–4, 33], *Escherichia* [2–4, 33], *Lactobacillus* [2–4, 33], and *Megasphaera* [34], and the negative associations of *Ruminococcus* [2–4, 33] and *Aldercreutzia* [4, 35]. Despite these consistencies, concerns over the potential confounding by antidiabetic treatments, especially metformin use, in characterizing diabetes-related gut microbiota have been raised [27–29, 36, 37]. Our results also indicated that associations of *Escherichia*, *Intestinibacter*, and possibly *Shigella*, with diabetes might be partially attributed to the effects of antidiabetic medication use. However, some evidence suggests that these gut bacteria may still play a role in host metabolic health and diseases [33, 38]. For example, *Intestinibacter* might be beneficial to host lipid and glucose metabolism and intestinal barrier integrity through its production of biomolecules such as branched SCFAs (isobutyrate and isovalerate) [39, 40].

In searching for potential mechanisms underlying the observed association between gut bacteria and diabetes, our results suggested that alterations in several circulating immune and proinflammatory proteins, especially IL-18R1 and OPG, might play a role. Notably, IL-18R1 is the receptor of proinflammatory cytokine IL-18, and overproduction of IL-18 has been noted as a proxy biomarker for overall inflammation and immune activation in HIV infection [41] and metabolic-inflammatory diseases (e.g., diabetes [42] and non-alcoholic fatty liver diseases (NAFLD) [43]). Moreover, hyperactive of IL-18/IL-18R1 signaling in intestinal epithelial cells has been linked to pathologic breakdown of intestinal mucosal barrier and gut dysbiosis [44]. The observed inverse associations of *Ruminococcus* and *Adlercreutzia* with IL-18R1 and diabetes thus might be related to the potential anti-inflammatory effects of these two bacteria given their functionalities in generating biomolecules with immunomodulation and inflammation suppressive effects (e.g., SCFAs from *Ruminococcus* species [45]; equol, an isoflavandiol estrogen, from *Adlercreutzia * [46]). Regarding OPG, it is known as an osteoclastogensis inhibitory factor, playing a critical role in bone metabolism through inhibiting the receptor activator of nuclear factor kappa-B (RANK)/RANK ligand (RANKL) pathway [47]. The interaction between RANK/RANKL/OPG axis and gut microbiota has been documented in osteoporosis [48–50], a common bone disorder coexisting with diabetes [51]. In line with our observations, overproduction of OPG has been associated with diabetes [52, 53] and metabolic dysfunction-associated [54] or non-alcoholic fatty liver disease [55]. This adds support that OPG signaling might be involved in the relationship between gut dysbiosis and metabolic complications beyond bone metabolism.

Our results also revealed that another five proteins (HGF, CD6, fibroblast growth factor 21 (FGF-21), LIF-R, and OSM) might mediate the associations of gut bacteria with diabetes. Of note, the contributions of HGF [56] and OSM [57] to insulin resistance and glucose impairment in obesity and diabetes have reported, and elevated levels of LIF-R have been noted in hepatic steatosis and MAFLD-associated gut dysbiosis ( [54]). However, the relationships between the other two proteins and diabetes remain controversial, especially for FGF21 [58]. While increased circulating FGF-21 levels was linked to elevated risk of diabetes [59], null results were reported in Mendelian Randomization investigations [60]. FGF-21 was report to enhance skeletal muscle glucose uptake [61] and protect lipid disruption and nonalcoholic steatohepatitis [60, 62], even in a clinical trial [63]. These data suggest that the increased circulating FGF21 in diabetes might be a compensatory mechanism to hyperglycemia or hyperlipidemia [61], echoed with observations regarding the elevated FGF21 levels in HIV infection [64]. More studies are required to reveal how these proteins and their interplay with gut microbiota contribute to diabetes and related metabolic complications. In addition, we found serval proteins associated with diabetes, including those previously reported (e.g., VEGFA [65], TWEAK [66], CXCL10 [67], and CXCL6 [68]), which were generally not associated with the identified diabetes-related gut bacteria, indicating that their associations with diabetes might not be related to the detected gut dysbiosis in our study.

Beside proteins, our results also suggested that alterations in metabolites, especially those microbial-derived, such as IMP and PAGln, might partially mediate the observed associations between gut bacteria and diabetes. Indeed, IMP is a well-characterized microbial metabolite generated from histidine catabolism [32] and has been linked to pathogenesis of impaired glucose metabolism and insulin resistance [32, 69]. In line with our results, increased abundances of many members in *Ruminococcus* and *Adlercreutzia* genera were associated with reduced IMP level [70], whereas *Escherichia*, *Intestinibacter*, and members in *Veillonellaceae* family (e.g., *Megasphaera*) were positively associated with IMP levels [70] as these bacteria harbor the urocanate reductases required for IMP production [32]. Similarly, PAGln is a microbiota derived metabolite from phenylalanine catabolism [31], originally being characterized as a risk factor of cardiovascular diseases [31, 71], and also has been linked to impaired glucose metabolism and diabetes [72]. Aligning with our observations, previous studies have demonstrated that the abundances of *Ruminococcus* and *Intestinibacter* species were inversely associated with blood PAGln [70]. Of note, the potential proinflammatory properties of IMP and PAGln have been documented in previous studies [31, 32, 69], and consistently, we also observed positive associations between these two microbial metabolites and multiple proinflammatory proteins (e.g., IL6 and IL17-A/C in Fig S 8). These findings further support the potential mediation effects of these metabolites and proinflammatory proteins in combination linking gut dysbiosis and diabetes. Additionally, our mediation analyses indicated that a lipid module characterized by high DGs but low CEs and several metabolites (e.g., alanine, dimethylguanidino valeric acid (DMGV), and xanthopterin) might partially explain the inverse associations of *Ruminococcus* and *Intestinibacter* with diabetes*,* and the positive association of *Shigella* with diabetes. However, whether and how the gut microbiota modulates the lipids (e.g., diradylglycerols and cholesterol esters) [73] and xenobiotics [74] remain to be further elucidated.

It has been proposed that there might be a triad relationship among HIV infection, gut dysbiosis, and diabetes [7, 75]. Although the identified diabetes-related gut bacteria were not associated with HIV and their associations with diabetes tended to be independent of HIV serostatus in this study, our further omics-wide association analyses identified multiple gut bacterial associated with HIV infection, with concordant associations with diabetes (e.g., *Lachnoclostridium* was positively associated with both HIV infection and diabetes, while *Faecalibactrium* was inversely associated with both HIV infection and diabetes). These results corroborate the idea that gut dysbiosis may be a crucial factor contributing to the compromised metabolic health in chronic HIV infection [7, 75]. Similarly, we also identified many metabolites and several proteins (e.g., SLAMF1: signaling lymphocytic activation molecule and IL-15RA: interleukin-15 receptor subunit alpha) associated with HIV infection and diabetes in the same direction. Furthermore, plasma levels of several proteins (e.g., IL-18R1, IL-6, and OPG), lipids (e.g., DGs/TGs), and nonlipid metabolites (e.g., 2-methylguanosine, xanthopterin, N4-acetylcytidine, glycocholic acid, PAGln, and IMP) that were positively associated with diabetes were elevated in PLWH in our and other studies [42, 76–78]. These observations collectively support the potential role of chronic inflammation/persistent immune activation and metabolic perturbations in bridging HIV infection with metabolic disorders [1].

Findings from this study have several implications. Modulation of the identified beneficial bacteria, such as *Ruminococcus* and *Adlercreutzia,* through supplementation or augmentation, could be explored as a potential intervention for diabetes prevention and management, especially among susceptible populations like PLWH given the observed anti-inflammatory properties of these bacteria in this study and existing literature [46, 79, 80]. As mechanisms of action for some microbial-derived metabolites become clearer, drugs inhibiting downstream signaling activation, such as deactivating or blocking p38gMAPK and mechanistic target of rapamycin complex 1 (mTORC1) signaling for IMP [32] and adrenergic receptors for PAGln [31], could present a novel and effective approach for diabetes prevention and treatment. Additionally, the identification of proteins mediating the connections between gut bacteria and diabetes support the concept of tailoring anti-inflammatory therapy for metabolic diseases, especially in the context of HIV infection [75, 81] [82]. Notably, drugs targeting the IL-18/IL-18R signaling axis, such as IL-18R antagonist or components resembling naturally occurring IL-18 binding protein (IL-18BP) and anti-IL-18 antibodies, are in development and testing for diabetes related conditions, including hypertension and chronic kidney diseases [83–85], as well as HIV infection [41]. Similarly, drugs like Denosumab targeting the RANKL/RANK/OPG pathway have been proposed as potential novel options for diabetes treatment and glycemic control [86, 87].

The main strength of this study is that we leveraged multi-omics data in a cohort of PLWH with demographically and socioeconomically comparable HIV seronegative participants. The validation of diabetes-associated metabolites with prospective analyses further solidifies our findings regarding the metabolic alterations in diabetes. Nevertheless, our study has several limitations. The nature of observation study limits causal inference, especially for the cross-sectional results of gut microbiota and proteins with diabetes. Findings need to be confirmed in prospective studies. Although we have carefully addressed the potential impacts of antidiabetic treatment on our results through “drug deconfounding” analysis, potential residual confounding related to varied types of medication use may still exist. Meanwhile, some potential confounding factors such as diet that have been associated with both gut microbiota and diabetes were not well addressed in the present study due to the lack of these data. Furthermore, the presented study was limited in the depth and coverage of omics techniques, especially for metabolites and proteins, which might partially prevent us further examining known molecules and pathways (e.g., gut microbial indole metabolites from tryptophan catabolism [20, 88]) as well as possibly novel ones, that are relevant to HIV infection and metabolic disorders. In addition, the relatively small number of HIV seronegative people limited our power to detect the effect modification by HIV infection. The associations of gut microbiota and serum proteins with diabetes were examined in women and need to be further validated in men and other HIV cohorts.

## Conclusions

In the present multi-omics analysis among individuals with and without HIV infection, we identified multiple gut bacteria, including two potentially beneficial genera (*Ruminococcus* and *Adlercreutzia*), numerous circulating metabolites, and proinflammatory proteins associated with diabetes. The observed gut bacteria–diabetes associations were partially explained by some microbial metabolites and proinflammatory proteins, supporting the role of gut microbiota in regulating host metabolite profile and inflammation/immune activation which may contribute to the development of diabetes.

### Supplementary Information

**Additional file 1: **Supplementary Methods. Additional descriptions on the drug deconfounding analysis and mediation analyses of multiple mediators.

**Additional file 2: Fig S1.** An overview of analysis and sample sizes; **Fig S2.** Partial spearman correlation (PCorr) among diabetes-associated gut bacteria genera and species within each genus; **Fig S3.** Results of ANCOM-II for prevalent diabetes at the species level; **Fig S4.** Confounding analysis of antidiabetic medications on selected diabetes-associated gut bacterial genera; **Fig S5.** Associations of metabolites with incident diabetes; **Fig S6.** Partial correlation (PCorr) among 76 diabetes-associated metabolites; **Fig S7.** Partial correlation among diabetes-associated individual lipids and four lipid modules; **Fig S8.** Partial correlation (PCorr) among diabetes-associated gut bacteria genera, nonlipid metabolites, lipid modules, and proteins; **Fig S9.** Relationship between associations of omics features with HIV positive status and associations with prevalent diabetes.

**Additional file 3: Table S1. **Population characteristics in 493 WIHS women.

**Additional file 4: Table S2. **The identification of Genera associated with prevalent diabetes in ANCOM-II.

** Additional file 5: Table S3. **Associations of selected gut bacteria genera with prevalent diabetes by adjustment models.

** Additional file 6: Table S4. **Associations of metabolites (and lipids modules) and proteins with diabetes by adjustment models.

** Additional file 7: Table S4. **Associations of metabolites (and lipids modules) and proteins with diabetes by adjustment models.

** Additional file 8: Table S6. **Associations of diabetes associated genera with diabetes associated metabolites (and Lipid modules) and proinflammatory proteins.

** Additional file 9: Table S7. **Associations of selected microbial genera, proteins, lipid modules and nonlipids metabolites with diabetes by HIV serostatus.

** Additional file 10: Table S8. **Levels of diabetes associated microbial genera, proteins, lipid modules and individual nonlipids metabolites by HIV characteristics.

** Additional file 11: Table S9. **HIV infection-associated gut bacteria, proteins and metabolites and prevalent diabetes in WIHS women.

## Data Availability

Access to individual-level data from the MACS/WIHS Combined Cohort Study Data (MWCCS) may be obtained upon review and requires an approval of a MWCCS concept sheet submission process. The timeline for the review and approval of any individual data applications may be varied but usually applicants will receive comments within 1 month. Links and instructions for online concept sheet submission and details on data sharing plan are on the study website (https://statepi.jhsph.edu/mwccs/work-with-us/).

## Abbreviations

HIV: Human immunodeficiency virus
PLWH: People living with HIV infection
ART: Antiretroviral therapy
SCFAs: Short-chain fatty acids
BCAAs: Branched-chain amino acids
COCOMO: The Copenhagen Comorbidity in HIV infection study
WIHS: The Women’s Interagency HIV Study
MACS: The Multicenter AIDS Cohort Study
ANCOM-II: The Analysis of Composition of Microbiome-II
FDR: False discovery rate
PERMANOVA: The permutational multivariate analysis of variance
BMI: Body mass index
SBP: Systolic blood pressure
HDL-C: High-density lipoprotein cholesterol
ANCOVA: Analysis of covariance
IL-18R1: Interleukin-18 receptor 1
CD6: T cell surface glycoprotein CD6 isoform
CXCL10: C-X-C motif chemokine 10
TWEAK: Tumor necrosis factor (ligand) superfamily, member 12
HGF: Hepatocyte growth factor
CSF-1: Macrophage colony-stimulating factor 1
OPG: Osteoprotegerin
VEGFA: Vascular endothelial growth factor A
GDNF: Glial cell line-derived neurotrophic factor
CARs: Carnitines
CEs: Cholesterol esters
PEs: Phosphatidylethanolamines
PCs: Phosphatidylcholines
LPCs: Lyso-phosphatidylcholines
TMAO: Trimethylamine N-oxide
PAGln: Phenylacetylglutamine
IMP: Imidazolepropionic acid
LIF-R: Leukemia inhibitory factor receptor
RANK: Receptor activator of nuclear factor kappa-B
FGF-21: Fibroblast growth factor 21
DMGV: Dimethylguanidino valeric acid
mTORC1: Mechanistic target of rapamycin complex 1
SLAMF1: Signaling lymphocytic activation molecule
IL-15RA: Interleukin-15 receptor subunit alpha
