# Supplementary material for: Metabolic and inflammatory perturbation of diabetes associated gut dysbiosis in people living with and without HIV infection
Source: Genome Med. 2024 Apr 20;16:59. doi: 10.1186/s13073-024-01336-1 (PMC11032597; doi:10.1186/s13073-024-01336-1)
Supplement: Supplementary file 1 — Additional file 1: Supplementary Methods. Additional descriptions on the drug deconfounding analysis and mediation analyses of multiple mediators. [file 13073_2024_1336_MOESM1_ESM.docx]

**Supplementary Methods**

**Metabolic and inflammatory perturbation of diabetes associated gut dysbiosis in people living with and without HIV infection**

Kai Luo^1^, Brandilyn A Peters^1^, Jee-Young Moon^1^, Xiaonan Xue^1^, Zheng Wang^1^, Mykhaylo Usyk^2^, David B Hanna^1^, Alan L Landay^3^, Michael F Schneider^4^, Deborah Gustafson^5^, Kathleen M Weber ^6^, Audrey French^3^, Anjali Sharma^7^, Kathryn Anastos^1,7,8^, Tao Wang^1^, Todd Brown^9^, Clary B Clish^10^, Robert C Kaplan^1,11^, Rob Knight^12,13,14,15^, Robert D Burk^1,2,8,16^, Qibin Qi^1,17*^

^1^Department of Epidemiology and Population Health, Albert Einstein College of Medicine, Bronx, NY 10461, USA

^2^ Department of Microbiology & Immunology, Albert Einstein College of Medicine, Bronx, New York, USA
^3^ Department of Internal Medicine, Rush University Medical Center, Chicago, IL, USA

^4^ Department of Epidemiology, Johns Hopkins Bloomberg School of Public Health, Baltimore, MD, USA
^5^ Department of Neurology, State University of New York-Downstate Medical Center, Brooklyn, New York, USA

^6^ Hektoen Institute of Medicine, Chicago, IL, USA
^7^ Department of Medicine, Albert Einstein College of Medicine, Bronx, New York, USA
^8^ Department of Obstetrics & Gynecology and Women’s Health, Albert Einstein College of Medicine, Bronx, New York, USA

^9^ Division of Endocrinology, Diabetes, and Metabolism, Department of Medicine, Johns Hopkins University School of Medicine, Baltimore, USA

^10^ Broad Institute of MIT and Harvard, Cambridge, MA, USA

^11^ Public Health Sciences Division, Fred Hutchinson Cancer Research Center, Seattle, WA, USA

^12^ Center for Microbiome Innovation, University of California, San Diego, La Jolla, CA, USA

^13^ Department of Bioengineering, University of California, San Diego, La Jolla, CA, USA

^14^ Department of Pediatrics, University of California, San Diego, La Jolla, CA, USA

^15^ Department of Computer Science and Engineering, University of California, San Diego, La Jolla, CA, USA

^16^ Department of Pediatrics, Albert Einstein College of Medicine, Bronx, New York, USA

^17^ Department of Nutrition, Harvard T.H. Chan School of Public Health, Boston, MA, USA

**Correspondence**: Dr. Qibin Qi (qibin.qi@einsteinmed.edu) at Department of Epidemiology and Population Health, Albert Einstein College of Medicine, 1300 Morris Park Avenue, Bronx, NY, 10461, USA

**Keywords**: HIV infection; diabetes; gut microbiota; metabolites; proteins; multi-omics

**Drug deconfounding analysis**

This “drug deconfounding” analysis was conducted using the R package “metadeconfoundR”[1]. A detailed description and graphic illustrations of this method can be found at <https://github.com/TillBirkner/metadeconfoundR>. The main analysis was performed using the Metadeconfound() function in the package in an intrinsic two-step process [1]. In the first, all associations between microbial features and the set of independent variables (diabetes, antidiabetic medications, and covariates [e.g., age, education]) are determined using non-parametric statistics (Mann–Whitney U-test (MWU) or Spearman test, adjusted for multiple testing using the Benjamini–Hochberg method). Each microbial feature significantly (FDR < 0.1) associated with diabetes is checked for associations with potential confounders. If not, it is considered trivially unconfounded (not confounded (NC)). If at least one covariate also has a significant association with microbial feature, then a post hoc test for confounding is applied to each such covariate. This test takes the form of comparing a nested linear model (likelihood ratio test (LRT) for *P* values), where the dependent variable is the microbial feature, and the independent variables are the diabetes status and tested covariate (e.g., antidiabetic medications use) versus a model containing only the covariate to whether diabetes adds explanatory statistical power beyond covariate. If this holds (*P* of LRT < 0.05) for all covariates, then diabetes is confidentially deconfounded (CD) concerning its association with microbial feature. For each covariate that loses significance, a complementary modeling test of the complementary model pairs is performed, predicting microbial feature as a function of diabetes status and covariate versus a model containing diabetes status alone. If for at least one such covariate, covariate has independent effect (*P* of LRT  < 0.05) on top of diabetes status, then the microbial feature is considered confounded by covariate. However, if in none of the pairwise tests the original significance holds, then diabetes status and covariate are considered so correlated that their relative influence cannot be disentangled. We consider these cases ambiguously deconfounded (AD), in the sense that clear confounding influence can neither be concluded nor ruled out.

[1] Forslund SK, Chakaroun R, Zimmermann-Kogadeeva M, et al. (2021) Combinatorial, additive and dose-dependent drug-microbiome associations. Nature 600(7889): 500-505. 10.1038/s41586-021-04177-9

**Mediation analyses with multiple mediators**

A counterfactual regression-based causal mediation was applied to assess the mediating effects of individual and multiple identified diabetes-associated omics features in the association between gut bacteria and diabetes. Methodology details were presented in [2, 3] and also can be found at <https://bs1125.github.io/CMAverse/>. Below are the formulas for the demonstrations of mediating effects of multiple mediators. For mediators vector M = ($M^{\left( 1 \right)}, \ldots,M^{\left( K \right)}$), the regression model for outcome can defined in below (1),

$E \left[ Y | a,m,c \right]=\theta_{0}+\theta_{1}a+\theta_{2}^{\left( 1 \right)}m^{\left( 1 \right)}+\theta_{2}^{\left( 2 \right)}m^{\left( 2 \right)}+\ldots+\theta_{2}^{\left( k \right)}m^{k}+\theta_{3}^{\left( k \right)}c \left( 1 \right)$, where logistic regression was used and Y, a, m, c refer to the outcome, exposure, mediator, and covariate, respectively. Regression models for mediators are defined in below (2): $E \left[ M^{(i)} | a,c \right]=\beta_{0}^{\left( i \right)}+\theta_{1}^{\left( i \right)}\alpha+\theta_{2}^{{(k)}^{'}}c for i=1,\ldots K \left( 2 \right)$, where multiple linear regression was used. Natural direct and indirect effects for a change in exposure from level $a^{*}$to level *a* are then given by: Natural direct effect (NDE)= $\theta_{1}\left( a-a^{*} \right)$ and Natural indirect effect (NIE)= $(\beta_{1}^{\left( 1 \right)}\theta_{2}^{\left( 1 \right)}+\ldots+\beta_{1}^{\left( K \right)}\theta_{2}^{\left( K \right)})\left( a-a^{*} \right)$*.*  In our study, $\left( a-a^{*} \right)$ was defined as 1 unit change from medians of exposures (i.e., the standardized centered log-ratio transformed bacteria abundance). The total effect (TE)=NDE+NIE. The proportion of mediating effect for multiple mediators is thus defined as ratio of NIE to TE. In practice, the “cmest” function from the R package “CMAverse” was used to get the estimates. A 20000 bootstrapping was applied to derive the 95% confidence intervals.

References:

[2] Valeri L, Vanderweele TJ: **Mediation analysis allowing for exposure-mediator interactions and causal interpretation: theoretical assumptions and implementation with SAS and SPSS macros.** *Psychol Methods* 2013, **18:**137-150.

[3] VanderWeele TJ, Vansteelandt S: **Mediation Analysis with Multiple Mediators.** *Epidemiol Methods* 2014, **2:**95-115.
