# Supplementary material for: Metabolic and inflammatory perturbation of diabetes associated gut dysbiosis in people living with and without HIV infection
Source: Genome Med. 2024 Apr 20;16:59. doi: 10.1186/s13073-024-01336-1 (PMC11032597; doi:10.1186/s13073-024-01336-1)
Supplement: Supplementary file 2 — Additional file 2: Fig S1. An overview of analysis and sample sizes; Fig S2. Partial spearman correlation (PCorr) among diabetes-associated gut bacteria genera and species within each genus; Fig S3. Results of ANCOM-II for prevalent diabetes at the species level; Fig S4. Confounding analysis of antidiabetic medications on selected diabetes-associated gut bacterial genera; Fig S5. Associations of metabolites with incident diabetes; Fig S6. Partial correlation (PCorr) among 76 diabetes-associated metabolites; Fig S7. Partial correlation among diabetes-associated individual lipids and four lipid modules; Fig S8. Partial correlation (PCorr) among diabetes-associated gut bacteria genera, nonlipid metabolites, lipid modules, and proteins; Fig S9. Relationship between associations of omics features with HIV positive status and associations with prevalent diabetes. [file 13073_2024_1336_MOESM2_ESM.docx]

**Supplementary Figures**

**Metabolic and inflammatory perturbation of diabetes associated gut dysbiosis in people living with and without HIV infection**

Kai Luo^1^, Brandilyn A Peters^1^, Jee-Young Moon^1^, Xiaonan Xue^1^, Zheng Wang^1^, Mykhaylo Usyk^2^, David B Hanna^1^, Alan L Landay^3^, Michael F Schneider^4^, Deborah Gustafson^5^, Kathleen M Weber ^6^, Audrey French^3^, Anjali Sharma^7^, Kathryn Anastos^1,7,8^, Tao Wang^1^, Todd Brown^9^, Clary B Clish^10^, Robert C Kaplan^1,11^, Rob Knight^12,13,14,15^, Robert D Burk^1,2,8,16^, Qibin Qi^1,17*^

^1^Department of Epidemiology and Population Health, Albert Einstein College of Medicine, Bronx, NY 10461, USA

^2^ Department of Microbiology & Immunology, Albert Einstein College of Medicine, Bronx, New York, USA
^3^ Department of Internal Medicine, Rush University Medical Center, Chicago, IL, USA

^4^ Department of Epidemiology, Johns Hopkins Bloomberg School of Public Health, Baltimore, MD, USA
^5^ Department of Neurology, State University of New York-Downstate Medical Center, Brooklyn, New York, USA

^6^ Hektoen Institute of Medicine, Chicago, IL, USA
^7^ Department of Medicine, Albert Einstein College of Medicine, Bronx, New York, USA
^8^ Department of Obstetrics & Gynecology and Women’s Health, Albert Einstein College of Medicine, Bronx, New York, USA

^9^ Division of Endocrinology, Diabetes, and Metabolism, Department of Medicine, Johns Hopkins University School of Medicine, Baltimore, USA

^10^ Broad Institute of MIT and Harvard, Cambridge, MA, USA

^11^ Public Health Sciences Division, Fred Hutchinson Cancer Research Center, Seattle, WA, USA

^12^ Center for Microbiome Innovation, University of California, San Diego, La Jolla, CA, USA

^13^ Department of Bioengineering, University of California, San Diego, La Jolla, CA, USA

^14^ Department of Pediatrics, University of California, San Diego, La Jolla, CA, USA

^15^ Department of Computer Science and Engineering, University of California, San Diego, La Jolla, CA, USA

^16^ Department of Pediatrics, Albert Einstein College of Medicine, Bronx, New York, USA

^17^ Department of Nutrition, Harvard T.H. Chan School of Public Health, Boston, MA, USA

**Correspondence**: Dr. Qibin Qi (qibin.qi@einsteinmed.edu) at Department of Epidemiology and Population Health, Albert Einstein College of Medicine, 1300 Morris Park Avenue, Bronx, NY, 10461, USA

**Keywords**: HIV infection; diabetes; gut microbiota; metabolites; proteins; multi-omics


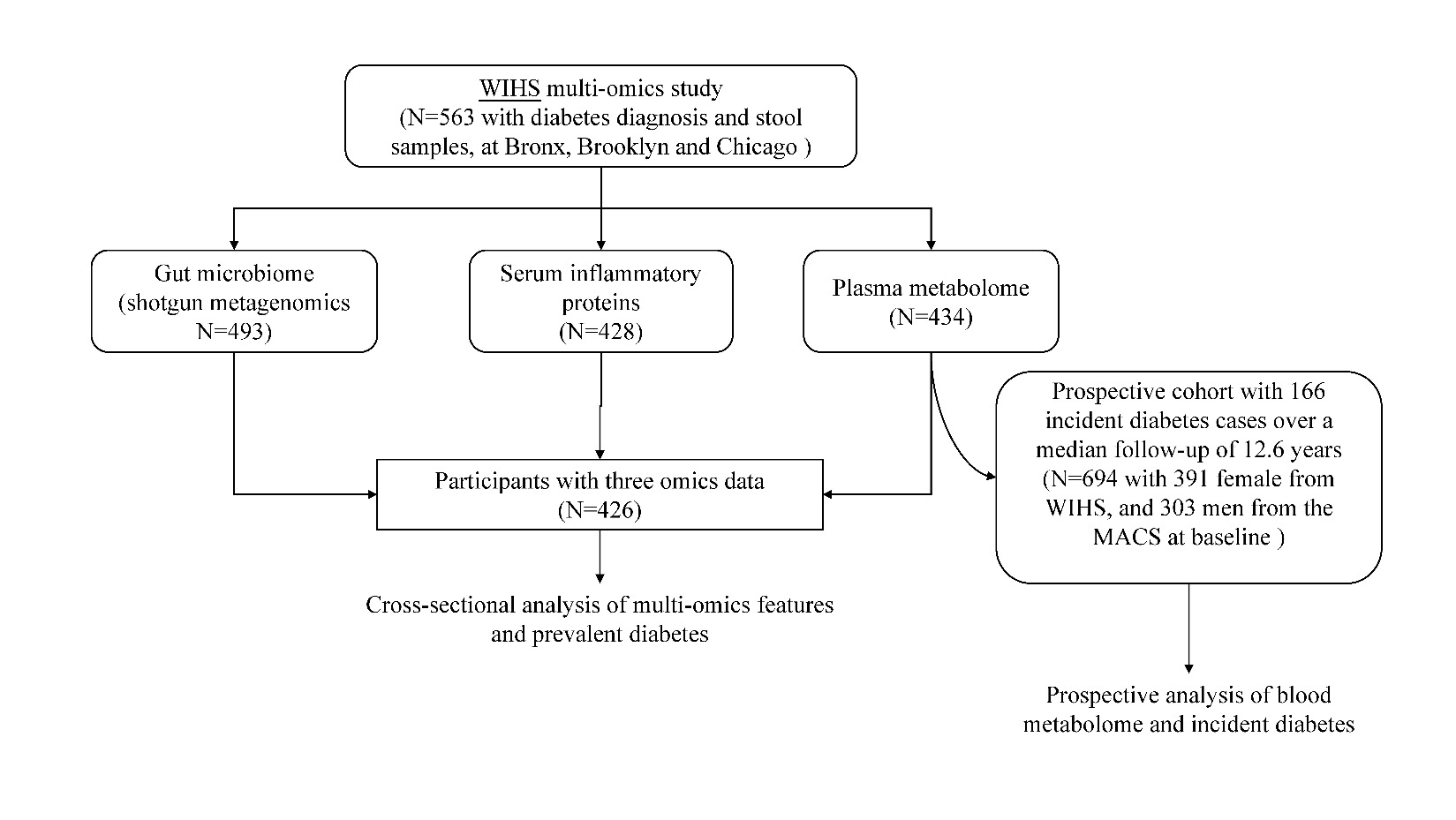


**Fig S1.** An overview of analysis and sample sizes. WIHS: Women’s Interagency HIV Study; MACS: Multicenter AIDS Cohort Study (MACS). In prospective analysis for incident diabetes, 694 participants were free of diabetes and with metabolomic and lipidomic data at a baseline visit (2004-2006) [1, 2].

[1]Zhang E, Chai JC, Deik AA, et al. (2021) Plasma Lipidomic Profiles and Risk of Diabetes: 2 Prospective Cohorts of HIV-Infected and HIV-Uninfected Individuals. J Clin Endocrinol Metab 106(4): 999-1010. 10.1210/clinem/dgab011

[2]Qi Q, Hua S, Clish CB, et al. (2018) Plasma Tryptophan-Kynurenine Metabolites Are Altered in Human Immunodeficiency Virus Infection and Associated With Progression of Carotid Artery Atherosclerosis. Clin Infect Dis 67(2): 235-242. 10.1093/cid/ciy053


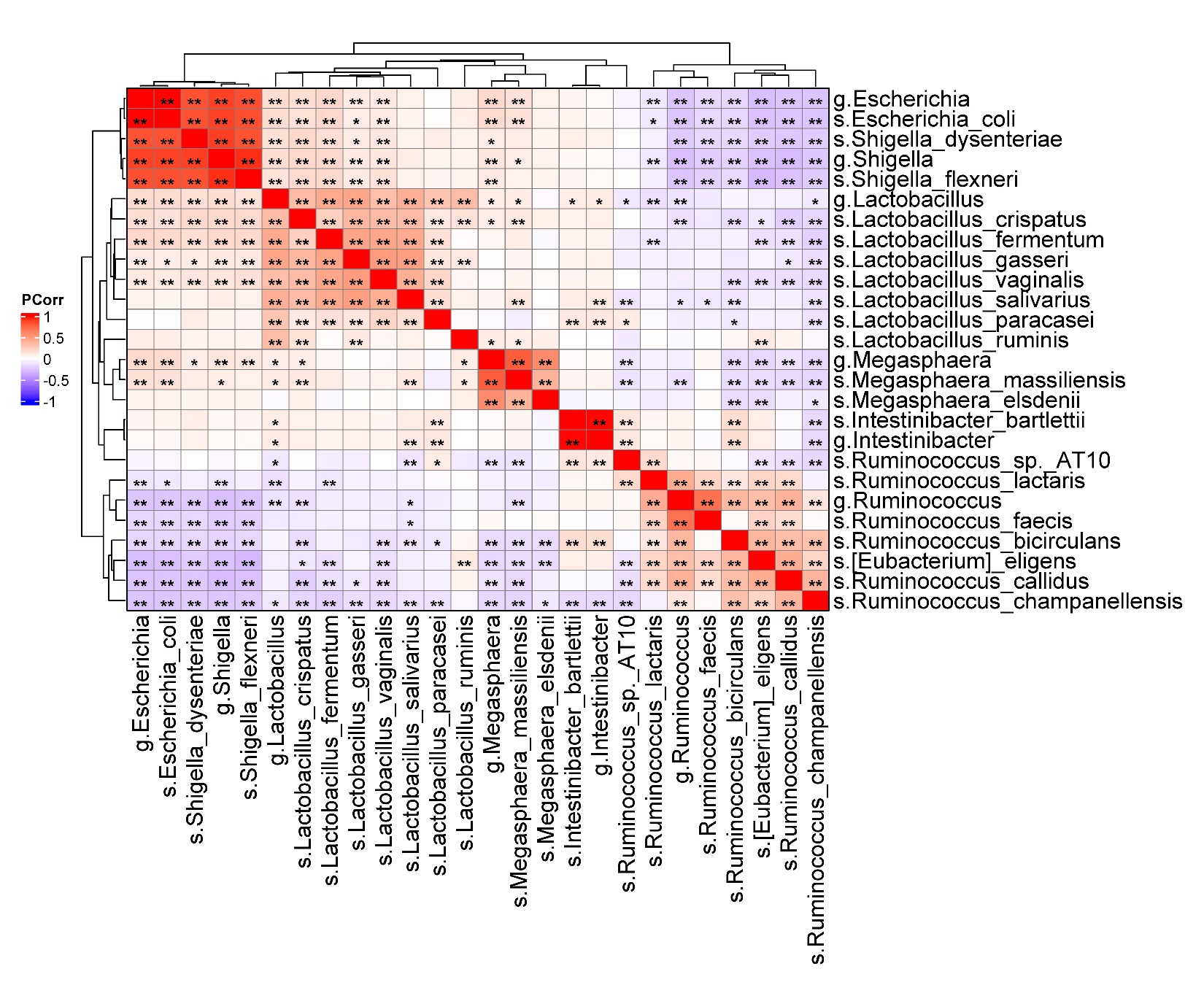


**Fig S2**. Partial spearman correlation (PCorr) among diabetes-associated gut bacteria genera and species (relative abundance >0.01% and detection rate >5%) within each genus. Abundances of taxa were central log ratio transformed and PCorr coefficients were adjusted for age at visit, study site, race/ethnicity, household annual income, education, smoking, alcohol consumption, HIV serostatus and antibiotic use within four weeks of stool sample collection. **FDR-q <0.001; * 0.001<FDR-q<0.05.


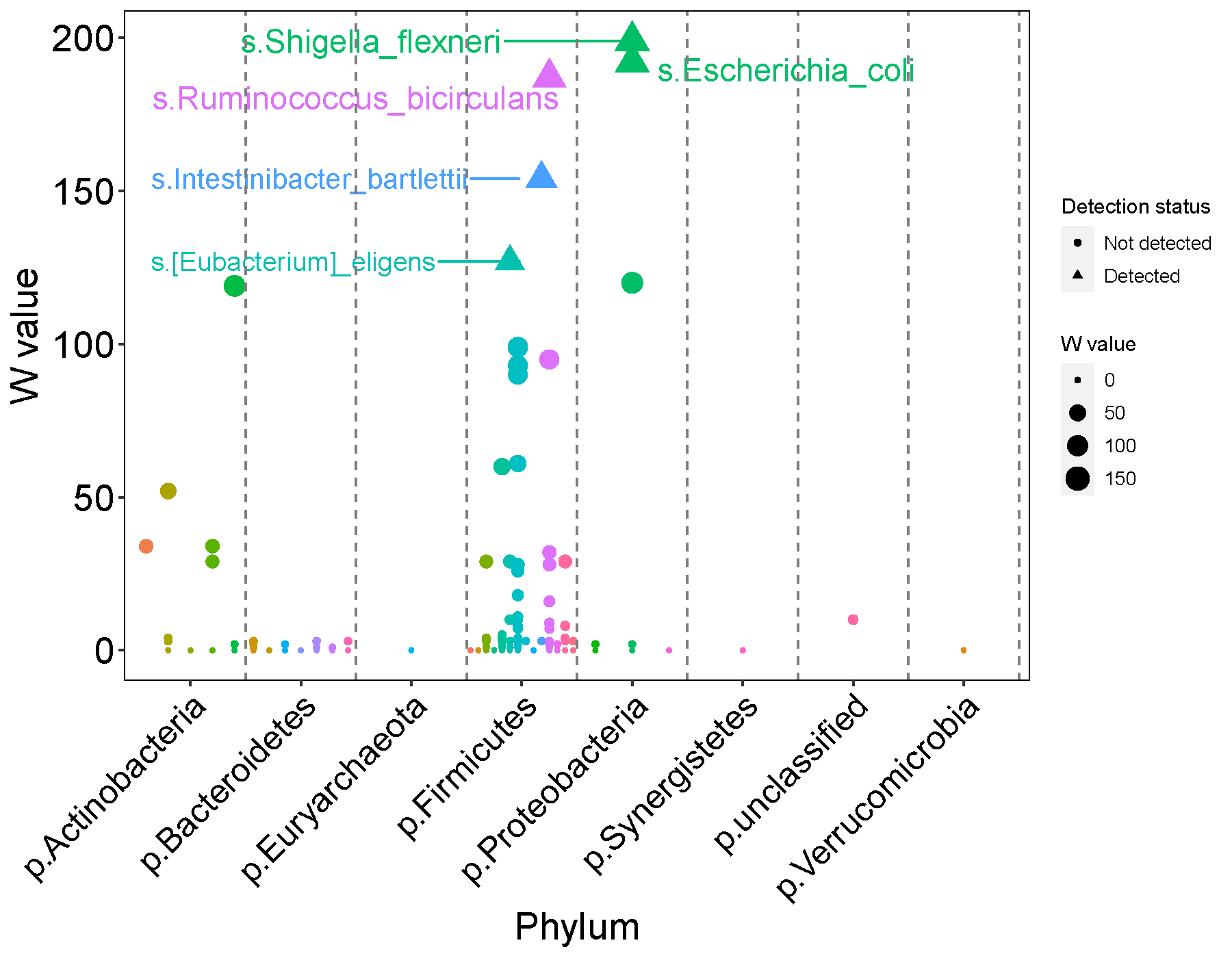


**Fig S3**. Results of ANCOM-II for prevalent diabetes at the species level (N=493). Distribution of W values of the 203 predominant microbiota species (relative abundance >0.01% and detection rate >10% of participants) in ANCOM-II, wherein age at visit, study site, race/ethnicity, household annual income, education, smoking, alcohol consumption status, HIV serostatus and antibiotic use within four weeks of stool sample collection were adjusted for. W values refer to the number of taxa with FDR-q values less than the selected threshold (0.10) in ANCOM-II. Species marked as triangles were those associated with diabetes with FDR-q <0.10 at threshold of 0.60 (i.e., the ratio of species to at least 60% of the other taxa is detected to be significantly associated with diabetes), wherein the colors and sizes refer to the class of phyla and W values in ANCOM-II.

.


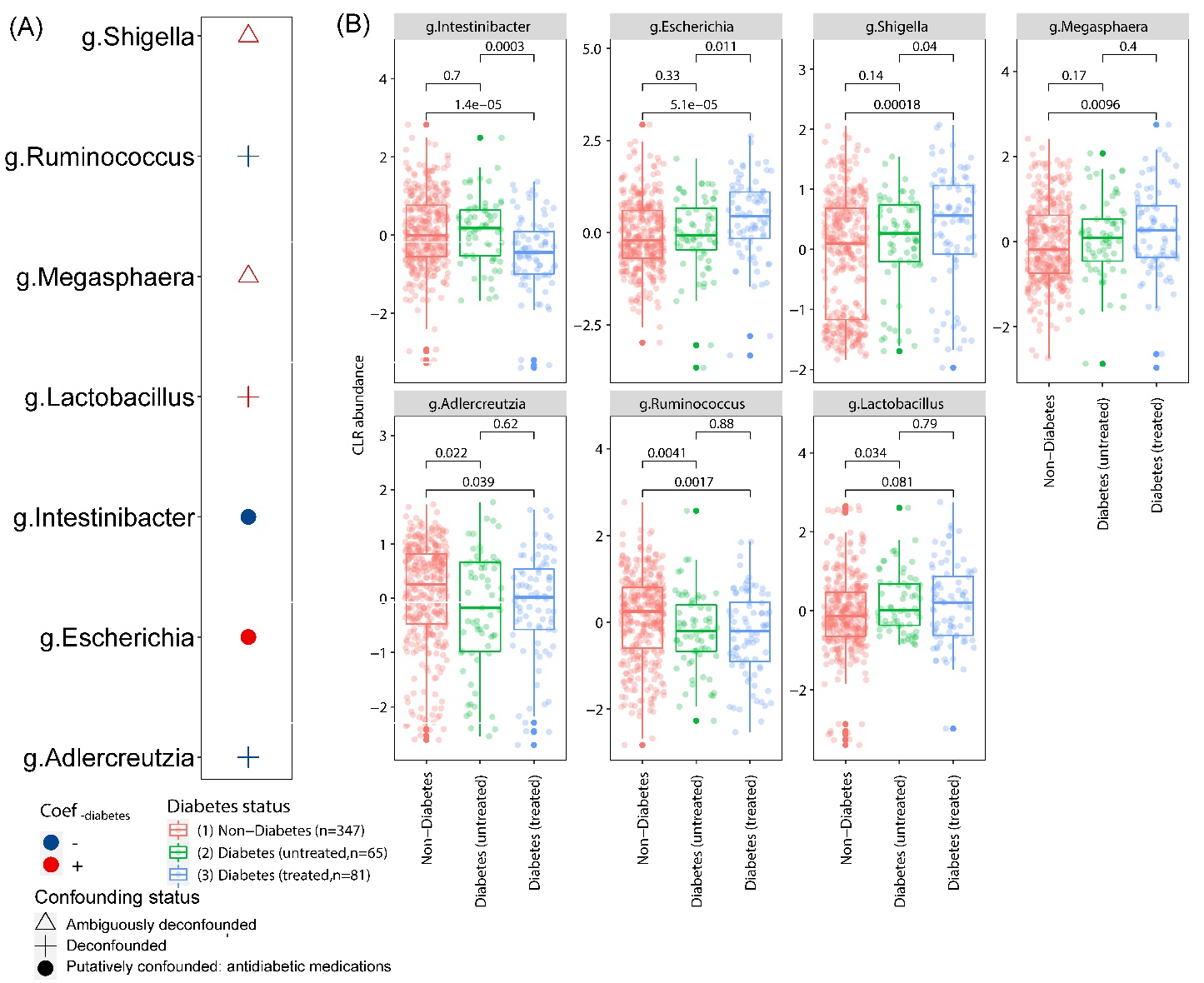


**Fig S4**. Confounding analysis of antidiabetic medications on selected diabetes-associated gut bacterial genera. **(A)** Results of confounding analysis for gut bacterial genera-diabetes associations. Confounding analysis was performed using the R package “MetadeconfoundR”[3]. Triangle, the sign of plus, and solid dot refers to the associations of the bacteria with diabetes tended to be ambiguously deconfounded, deconfounded, and putatively confounded by anti-diabetic medications as described in abovementioned “Drug deconfounding analysis” section. **(B)** The distribution of the central log ratio-transformed abundance of 7 diabetes-associated genera across non-diabetic participants and participants with and without antidiabetic treatments. Differences of genera abundances across diabetes groups were compared in ANOVA, adjusting for age at visit, study site, race/ethnicity, household annual income, education, smoking, alcohol consumption status, and antibiotic use.

[3] Forslund SK, Chakaroun R, Zimmermann-Kogadeeva M, et al. (2021) Combinatorial, additive and dose-dependent drug-microbiome associations. Nature 600(7889): 500-505. 10.1038/s41586-021-04177-9


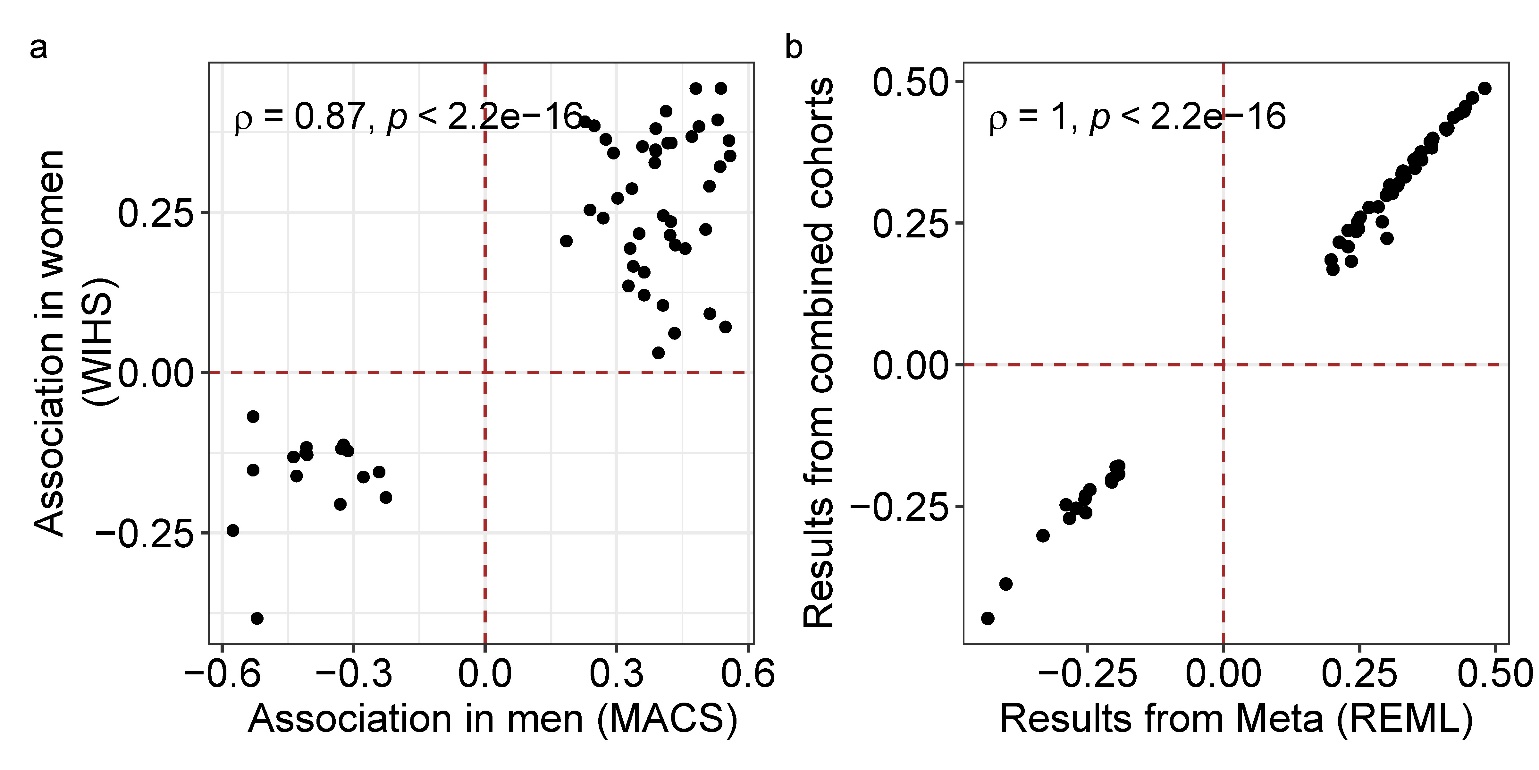


**Fig S5**. Associations of metabolites with incident diabetes. (a) results in WIHS women and MACS men. (b) correlation between estimates derived in combined cohorts and random-effect meta-analysis (REML). All estimates were derived while adjusting for age at visit, sex (only for analyses in combined cohorts), study site, race/ethnicity, smoking, alcohol consumption, annual household income, HIV serostatus. Detailed results were listed in Table S5.


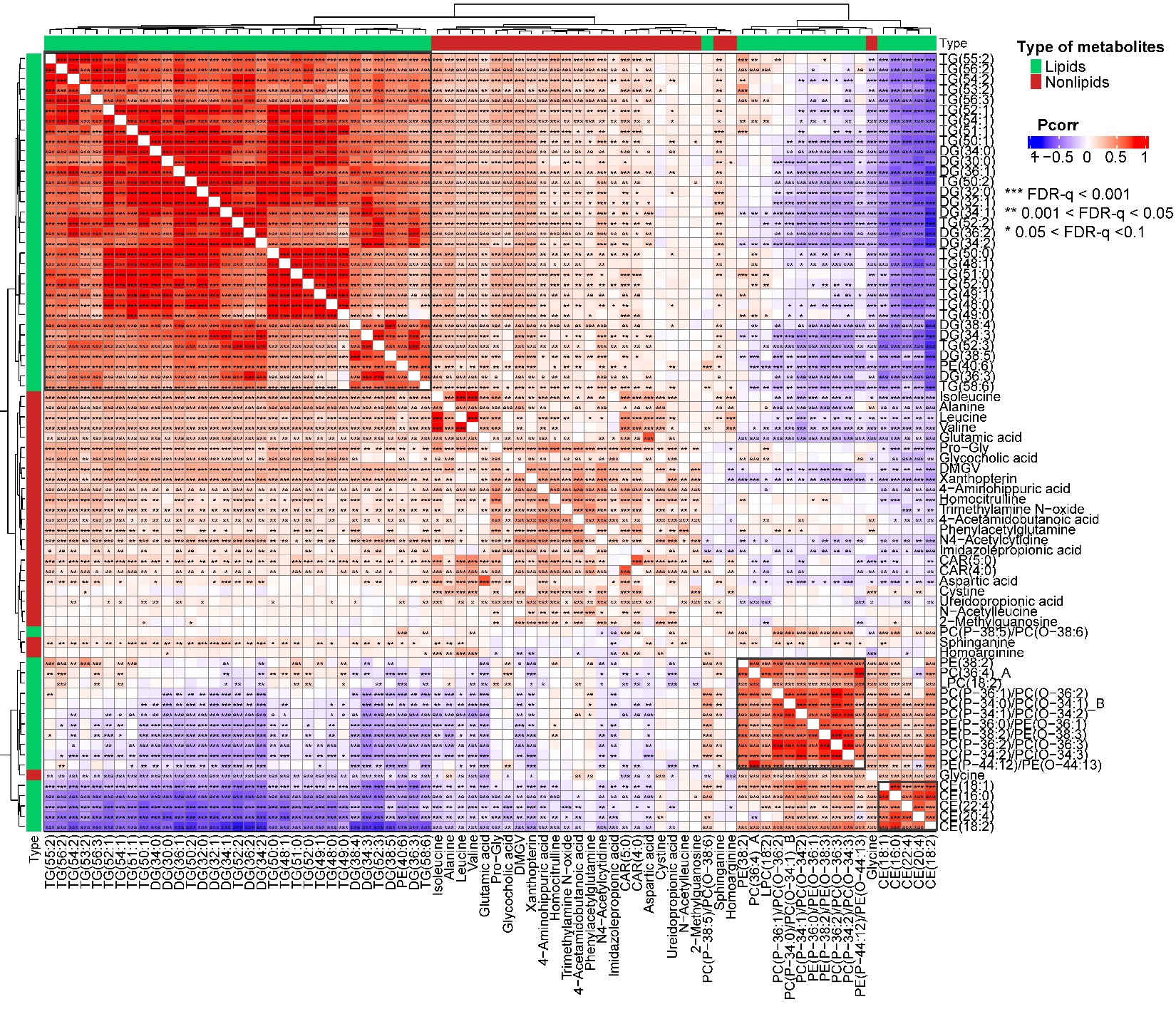


**Fig S6**. Partial correlation (PCorr) among 76 diabetes-associated metabolites. PCorr coeficients were adjusted for age at visit, study site, race/ethnicity, household annual income, education, smoking, alcohol consumption, HIV serostatus and antibiotic use within four weeks of stool sample collection. “Grey” frames show the correlation structure among lipids.


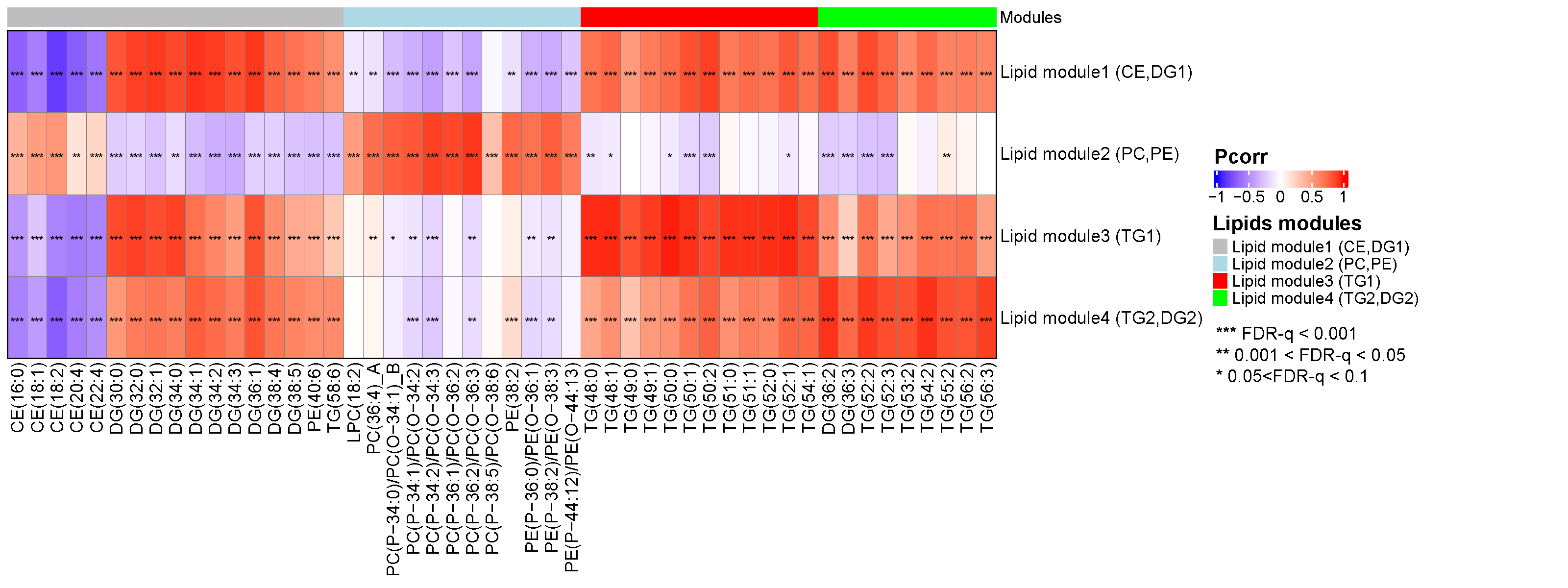


**Fig S7**. Partial correlation among diabetes-associated individual lipids (n=50) and four lipid modules. Estimates were adjusted for age at visit, study site, race/ethnicity, household annual income, education, smoking, alcohol consumption, HIV serostatus and antibiotic use within four weeks of stool sample collection.


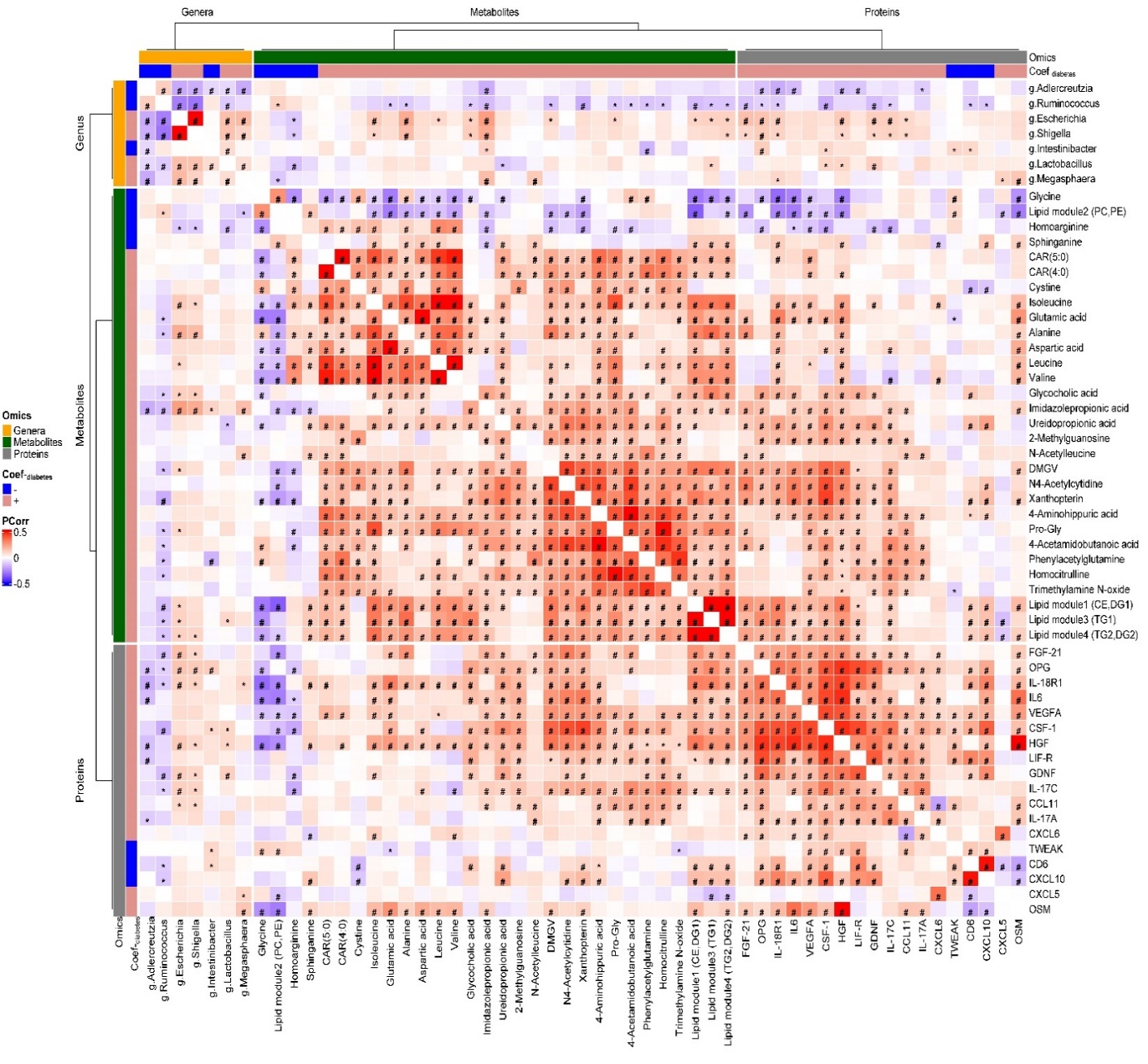


**Fig S8**. Partial correlation (PCorr) among diabetes-associated gut bacteria genera, nonlipid metabolites, lipid modules, and proteins. PCorr coefficients were adjusted for age at visit, study site, race/ethnicity, household annual income, education, smoking, alcohol consumption, HIV serostatus and antibiotic use within four weeks of stool sample collection. # FDR-q < 0.1,* *p* < 0.05. Full names of identified proteins were listed in Table S4.


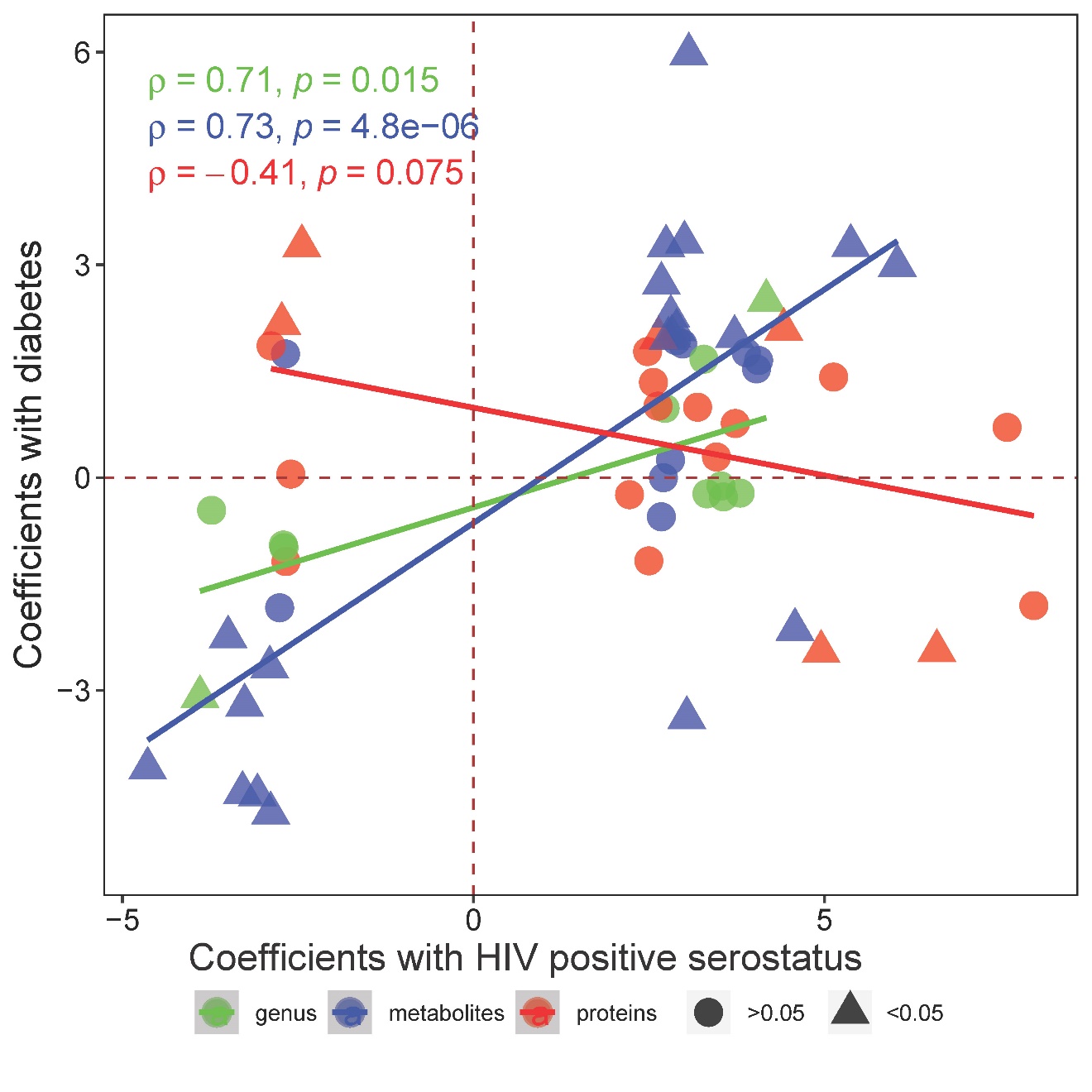


**Fig S9.** Relationship between associations of omics features with HIV positive status and associations with prevalent diabetes. Only omics features significantly associated with HIV positive status at FDR<0.1 were included. Associations were adjusted for age at visit, race/ethnicity, study sites, education, smoking, alcohol consumption, annual household income, fasting status, and HIV serostatus (only for associations with diabetes) among426 participants with proteins, metabolites, and bacteria genera data.
